# Synthesis of polyhydroxylated decalins via two consecutive one-pot reactions: 1,4-addition/aldol reaction followed by RCM/*syn*-dihydroxylation

**DOI:** 10.3762/bjoc.12.255

**Published:** 2016-12-01

**Authors:** Michał Malik, Sławomir Jarosz

**Affiliations:** 1 Institute of Organic Chemistry, Polish Academy of Sciences, Kasprzaka 44/52, 01-224 Warsaw, Poland

**Keywords:** carbasugars, one-pot reactions, ring-closing metathesis, *syn*-dihydroxylation

## Abstract

Synthesis of novel polyhydroxylated derivatives of decalin is described. The presented methodology consists in a one-pot copper-catalyzed 1,4-addition of vinylmagnesium bromide to sugar-derived cyclohexenone, followed by an aldol reaction with a derivative of but-3-enal. The obtained diene is then subjected to an assisted tandem catalytic sequence: ring-closing metathesis with the subsequent reuse of the Ru-catalyst in the *syn*-dihydroxylation. The stereochemical outcome of these reactions is discussed.

## Introduction

Derivatives of carbohydrates, in which the endocyclic oxygen atom is replaced with a methylene group are known as carbasugars [1]. Due to their similarity to carbohydrates, these compounds often possess interesting biological properties, e.g., they may act as glycosidase inhibitors [2–4]. One of such compounds, acarbose [5–7], found an application as a drug and is marketed worldwide.

**Figure 1:**
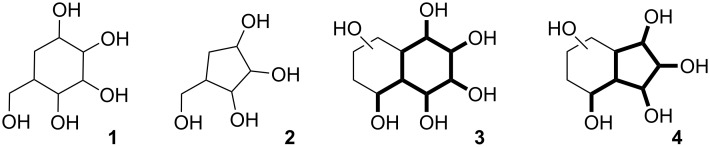
General structures of mono- and bicyclic carbasugars.

Five- and six-membered cyclitols are a well-known and recognizable group of carbasugars. The synthesis of these monocyclic compounds (such as **1** and **2**, Figure 1) and evaluation of their biological properties remain a challenging and vital research area [8–11].

On the other hand, bicyclic analogs of cyclitols are not as well-documented as their monocylic counterparts. However, polyhydroxylated derivatives of decalin and hydrindane are interesting synthetic targets, since they can be regarded as conformationally locked carbasugars (**3** and **4**, Figure 1) [12–15]. Indeed, some of the bicyclic carbasugars were proven to possess strong and selective anti-glycosidase activity [16–18]. The polyoxygenated bicyclic motif is also found in some members of the nargenicin antibiotics family [19–21].

**Scheme 1:**
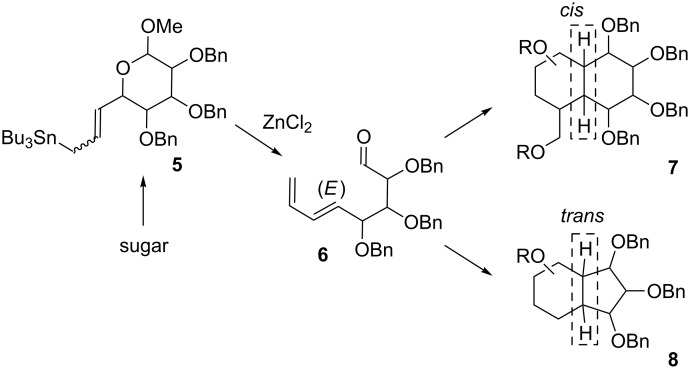
Approach to the synthesis of bicyclic carbasugars based on the use of sugar allyltins (previous works).

In our group, we have already proposed an approach to the synthesis of polyoxygenated bicyclic systems such as *cis*-decalins and *trans*-hydrindanes (Scheme 1) [22–25].

It relies on the use of sugar-derived allyltin (such as **5**) followed by ZnCl_2_-induced fragmentation into dienoaldehyde **6**, subsequent Horner–Wadsworth–Emmons olefination, and intramolecular Diels–Alder reaction.

**Scheme 2:**
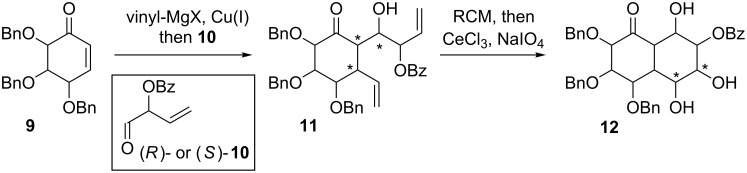
Approach to the synthesis of bicyclic decalins based on a 1,4-addition/aldol reaction followed by RCM with subsequent *syn*-dihydroxylation (this work).

In this paper, we explore the possibility of using a copper-catalyzed one-pot 1,4-addition of vinylmagnesium bromide to sugar-derived cyclic α,β-unsaturated ketones (such as **9**), followed by an aldol reaction with an optically pure derivative of but-2-enal **10** to obtain a mixture of diastereomeric dienes, such as **11** (Scheme 2).

As a result of this transformation, three new stereogenic centers are generated. A copper-catalyzed 1,4-addition of organometallic reagents to cyclic α,β-unsaturated ketones, followed by an aldol reaction has been already used in the synthesis of complex natural products [26–33]. Although this approach is challenging in terms of diastereoselectivity (up to eight possible diastereoisomers), it enables a rapid increase of molecular complexity. The major diastereoisomer of **11** can be then subjected to the assisted tandem catalytic sequence: RCM reaction, followed by the reuse of the ruthenium catalyst in the subsequent *syn*-dihydroxylation. As a result, the polyhydroxylated decalin derivative **12** was obtained. The s*yn*-dihydroxylation of cyclic alkenes is among the most widely used methods for the introduction of hydroxy groups onto the ring. Such transformations are usually conducted with osmium-based catalysts [34–35], but due to their high cost and toxicity, other methodologies (in vast majority based on ruthenium) were also proposed during the last years [36–40]. An interesting approach, based on a ruthenium catalysis, was independently proposed by Blechert and Snapper [41–42]. Both groups described a methodology, in which the ring-closing metathesis (RCM) reaction is followed by the reuse of the Ru catalyst in the *syn*-dihydroxylation step. In our recent papers [43–44], we extended this concise and effective approach to the synthesis of bicyclic iminosugars.

To our knowledge, a sequence of a one-pot 1,4-addition/aldol reaction and RCM/*syn*-dihydroxylation has not been used yet (in such a consecutive manner) for the preparation of highly functionalized bicyclic structures.

## Results and Discussion

**Scheme 3:**
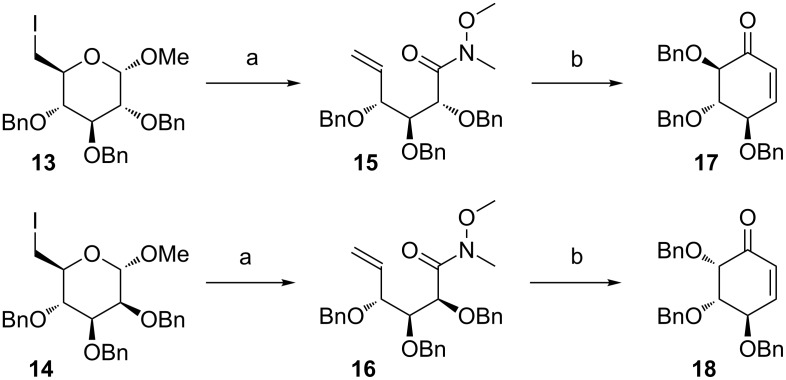
Reagents and conditions: (a) i. Zn, MeOH/H_2_O, 60 °C, 2 h, ii. Jones reagent, acetone, rt, 1 h, iii. MeNHOMe∙HCl, py, Ph_3_P, CBr_4_, rt, 24 h, 67% (**15**, 3 steps) or 68% (**16**, 3 steps); (b) i. vinyl-MgBr, THF, 0 °C, 1 h, ii. Hoveyda–Grubbs II cat. (5 mol %), toluene, 50 °C, 24 h, 61% (**17**, 2 steps) or 75% (**18**, 2 steps).

The synthesis of polyhydroxylated cyclohexenones was initiated from known iododerivatives **13** and **14**, prepared from suitably protected carbohydrates (Scheme 3) [45–47]. Each of these compounds was subjected to the zinc-mediated fragmentation, followed by the Jones oxidation.

The resulting acid (not purified) was transformed into a Weinreb amide. As a result of this three-step sequence of reactions, derivatives **15** and **16** were formed, respectively. Addition of vinylmagnesium bromide to **15** and **16** afforded the corresponding dienes, which – without further purification – were subjected to the RCM reaction with the Hoveyda–Grubbs II generation catalyst. As a result, derivatives **17** and **18** were obtained, respectively.

**Scheme 4:**
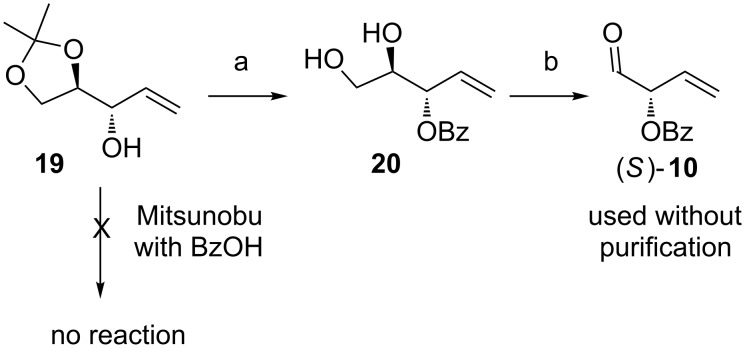
Reagents and conditions: (a) i. BzCl, DCM, Et_3_N, DMAP, rt, 24 h, ii. HCl, MeOH/H_2_O, rt, 24 h, 55% (2 steps); (b) NaIO_4_, NaHCO_3_, DCM/H_2_O.

The synthesis of aldehyde (*S*)-**10** was initiated from D-mannitol. A known procedure led to compound **19** [48], which was easily transformed into diol **20**, a convenient precursor of aldehyde (*S*)-**10** (Scheme 4). Since such an aldehyde would be most likely unstable, we decided to deprotect **20** directly before the planned one-pot procedure and use it without purification.

**Scheme 5:**
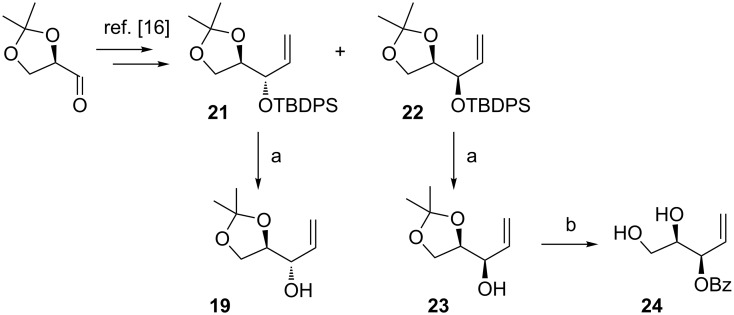
Reagents and conditions: (a) TBAF∙3H_2_O, THF, rt, 24 h, 96% (**19**) or 94% (**23**); (b) i. BzCl, DCM, Et_3_N, DMAP, rt, 24 h, ii. HCl, MeOH/H_2_O, rt, 24 h, 58% (2 steps).

In order to obtain (*R*)-**10**, we decided to inverse the configuration at the free hydroxy group in allylic alcohol **19** by Mitsunobu reaction. Despite the fact that the use of allylic alcohols as substrates in Mitsunobu reactions can be a challenging task, some successful examples can be found in the literature [49–50]. However, the reaction with benzoic acid did not proceed at all, whereas application of *p*-nitrobenzoic acid yielded a complicated mixture of products. Therefore, an alternative approach had to be used to obtain (*R*)-**10**. A known procedure based on addition of vinylmagnesium bromide to isopropylidene-protected D-glyceraldehyde followed by silylation and separation of an equimolar mixture of diastereoisomers led to optically pure olefins **21** and **22** (Scheme 5) [51]. After desilylation followed by protection of the free hydroxy with a benzoyl group and deprotection of the isopropylidene moiety, diols **20** and **24** were obtained.

**Scheme 6:**
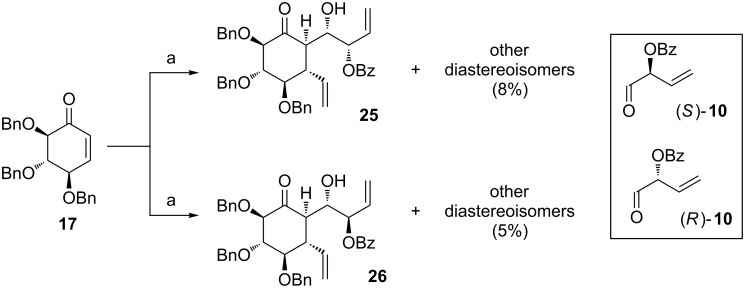
Reagents and conditions: (a) vinyl-MgBr, CuBr∙Me_2_S, THF, −45 °C, 15 min, then (*S*)- or (*R*)-**10**, −45 °C to −20 °C, 15 min, 54% (**25**) or 48% (**26**).

With α,β-unsaturated ketones **17** and **18** and two enantiomeric, optically pure aldehydes (*R*)- and (*S*)-**10** in hand, we started to study the 1,4-addition of vinyl-MgBr followed by an aldol reaction (Scheme 6).

**Figure 2:**
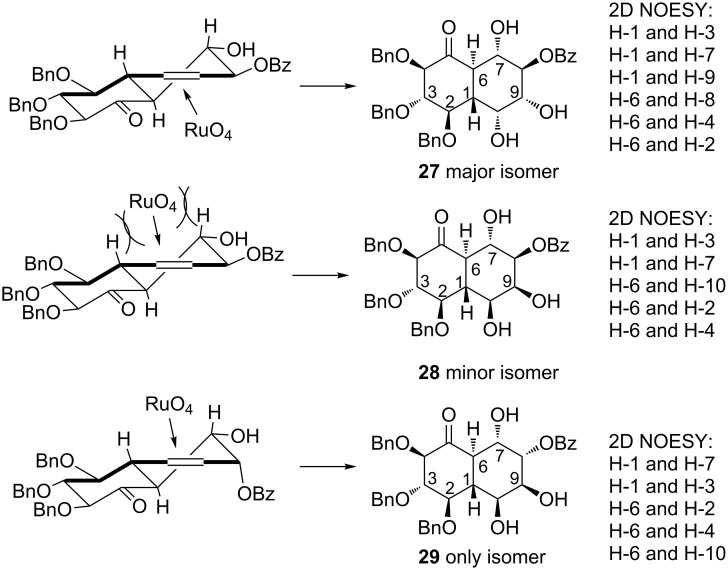
Possible course of the *syn*-dihydroxylation leading to **27**, **28**, and **29**.

The addition of a Grignard reagent to **17** was performed in THF at −45 °C in the presence of CuBr∙Me_2_S (0.5 equiv). After 15 min, a solution of aldehyde (either (*R*)- or (*S*)-**10**) in THF was added; the reaction mixture was warmed to −20 °C, and quenched with sat. NH_4_Cl. In the case of (*S*)-**10**, diolefin **25** was separated as major product (54%) out of 8 possible diastereoisomers. Moreover, small amounts of other diastereoisomers (ca. 8%) were also isolated as a complicated mixture. In the case of (*R*)-**10**, diolefin **26** was formed as major isomer (48%, contaminated with small amounts of other diastereoisomers of unknown stereochemistry). Small amounts of other isomers (ca. 5%) were also isolated as a complicated mixture. In both reactions, small amounts (ca. 10%) of intermediary product of 1,4-addition was also isolated. The stereochemistry of the obtained products was determined at the later stage (Figure 2). It turned out, that the transformation proceeds similarly in case of both enantiomers of **10**, regardless of the stereochemistry at the α-position. First, the addition of the vinyl-MgBr to **17** proceeded *anti* in relation to the neighbouring benzyloxy group. Then, the resulting enolate was approached by an aldehyde from the opposite side to the newly attached vinyl group. A similar stereochemical outcome of such transformation has been already reported [26–33]. Moreover, the addition of aldehyde gave rise to the *anti* aldol product (both in **25** and **26**). This is in accordance with the general observation, that *E*-enolates (enolates in six-membered rings are always *E*) give predominantly the *anti*-products in aldol reactions (Zimmerman–Traxler model) [52–54].

The application of the above-described procedure to cyclohexenone **18** was not successful, since it yielded a complicated, inseparable mixture of diastereoisomers instead of one major product.

**Scheme 7:**
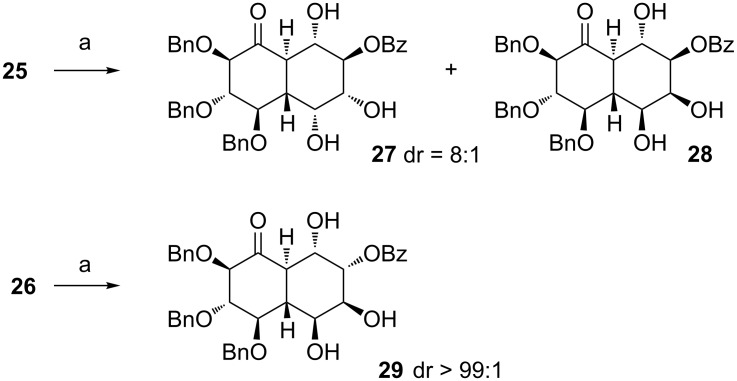
Reagents and conditions: (a) Hoveyda–Grubbs II cat. (5 mol %), toluene, 50 °C, 2 h, then evaporation, then NaIO_4_, CeCl_3_∙7H_2_O (15 mol %), MeCN/AcOEt, 0 °C, 20 min, 56% (**27** + **28**, dr = 8:1) or 53% (**29**, dr >99:1).

Both diolefins **25** and **26** were subjected to the ring-closing metathesis (Scheme 7) with the Hoveyda–Grubbs II generation catalyst (5 mol %), followed by the reuse of the Ru catalyst in the subsequent *syn*-dihydroxylation with NaIO_4_ as a stoichiometric oxidant in the presence of catalytic amounts of CeCl_3_ (15 mol %).

As a result, in the case of diolefin **25** as substrate, a separable mixture of diols (dr = 8:1; **27** being the major isomer) was obtained in fair yield (56%). The same procedure applied to compound **26** resulted in the formation of virtually one diastereoisomer **29** (53%). The structure of all compounds was assigned by 2D-NOESY experiments (Figure 2). In the case of both olefins, *syn*-dihydroxylation proceeded from the less congested face of the double bond (*anti* in the relation to the neighbouring benzoyl group).

**Scheme 8:**
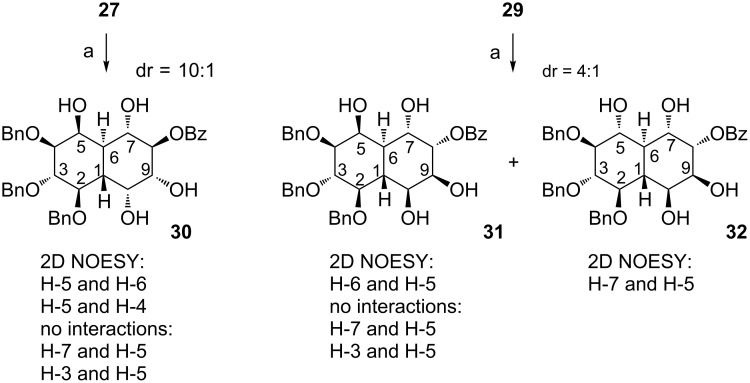
Reagents and conditions: (a) NaBH(OAc)_3_, MeCN/THF/AcOH, rt, 24 h, 67% (**30**, dr >99:1) or 74% (**31** + **32**, dr = 4:1).

Compounds **27** and **29** were subjected to Evans–Saksena reduction with NaBH(OAc)_3_ in MeCN/THF in the presence of AcOH (Scheme 8). Under these conditions, derivative **30** was obtained from **27** in good yield (67%) and with high diastereoselectivity (dr = 10:1; inseparable mixture). The same procedure applied to **29** yielded a separable mixture of diastereoisomers (dr = 4:1; **31** being the major isomer) in good yield (74%). The structure of all these compounds was confirmed by 2D-NOESY experiments (Scheme 8). In case of both substrates (**27** and **29**), the reduction proceeded *anti* in the relation to the β-hydroxy group [55–56]. These observations were confirmed by the NMR data in which proton H-4 (doublet of doublets) in **30** has one large coupling constant (9.5 Hz with H-3) and one small with H-5 (2.8 Hz), which indicates the *syn* relation between H-4 and H-5. Similar analysis in decalin **31** also points at the *syn* relation of H-4 and H-5. On the other hand, proton H-4 in **32** has two large coupling constants (~9.5 Hz), which indicates the *anti* relation between H-4 and H-5.

## Conclusion

We have demonstrated a convenient and simple route to optically pure polyhydroxylated *trans*-decalins. The methodology is based on two consecutive one-pot procedures. The first one consists in Cu(I)-catalyzed 1,4-addition of vinylmagnesium bromide followed by an aldol reaction, which is then followed by an assisted tandem catalytic sequence: Ru-catalyzed ring-closing metathesis with subsequent *syn*-dihydroxylation with the re-use of Ru catalyst. These transformations applied to optically pure 4,5,6-tribenzyloxycyclohexenone **17** derived from D-glucose gave the desired bicyclic structures in good yields and very good diastereoselectivities. To sum up, the proposed methodology is an efficient way for the preparation of highly oxygenated *trans*-decalins. It offers a possibility to significantly increase molecular complexity in just two steps.

## Supporting Information

File 1 Experimental procedures, spectral data, and copies of the ^1^H and ^13^C spectra for all new compounds.
