# Supplementary material for: Synthesis of polyhydroxylated decalins via two consecutive one-pot reactions: 1,4-addition/aldol reaction followed by RCM/syn-dihydroxylation
Source: Beilstein J Org Chem. 2016 Dec 1;12:2602–8. doi: 10.3762/bjoc.12.255 (PMC5238570; doi:10.3762/bjoc.12.255)

Supporting Information

for

Synthesis of polyhydroxylated decalins via two consecutive one-pot reactions: 1,4-addition/aldol reaction followed by RCM/*syn*-dihydroxylation

Michał Malik* and Sławomir Jarosz*

Address: Institute of Organic Chemistry, Polish Academy of Sciences, Kasprzaka 44/52, 01-224 Warsaw, Poland

Email: Michał Malik - michal.malik@icho.edu.pl; Sławomir Jarosz - slawomir.jarosz@icho.edu.pl

*Corresponding author

Experimental procedures, spectral data, and copies of the ^{1}H and ^{13}C spectra for all new compounds

Table of contents

Experimental ……….………………………………………………………………………….S1

Spectra …..……………………………………………………………………………………..S9

Experimental

NMR spectra were recorded in $CDCl_3$ with a Varian AM-600 (600 MHz ^{1}H, 150 MHz ^{13}C) or with a Varian AM-500 (500 MHz ^{1}H, 125 MHz ^{13}C) at room temperature. Chemical shifts (δ) are reported in ppm relative to TMS (δ 0.00) for ^{1}H and residual chloroform (δ 77.0) for ^{13}C. All resonances were assigned by COSY (^{1}H-^{1}H), HSQC (^{1}H-^{13}C) and HMBC (^{1}H-^{13}C) correlations. Relative stereochemistry was assigned based on the 2D-NOESY experiments. Mass spectra were recorded with a MALDI Synapt G2-S HDMS, melting points were measured with an SRS OptiMelt and are uncorrected. Optical rotations were measured with a Jasco P 1020 apparatus using sodium light (*c* 1, *T* = 23 ˚C; CH_2Cl_2). Elemental analyses were performed with an Elementar vario ELIII analyser. Reagents were purchased from Sigma-Aldrich, Alfa Aesar or ABCR, and were used without further purification. Dry solvents were purchased from Sigma-Aldrich and used as obtained. Organic solutions were dried over anhydrous $MgSO_4$ and concentrated under reduced pressure. Flash chromatography was performed on Grace Resolv cartridges, using a Grace Reveleris X2 system (UV and ELSD detection; gradient elution). Analytical and preparative TLC was performed on Silica Gel 60 F_{254} (Merck).

Synthesis of Weinreb amides

To the solution of iododerivative **13** or **14** (2.87 g, 5 mmol) in MeOH (50 mL), freshly activated zinc powder (15 g, 45 equiv) was added and the resulting mixture was stirred for 2 h at 60 °C. Then, the mixture was filtered through a pad of Celite which was then washed with MeOH (30 mL). The filtrate was concentrated to ca. 20 mL, after which water (50 mL) and AcOEt (100 mL) were added, the layers were separated, and the aqueous one washed with AcOEt (50 mL). Combined organic layers were dried, concentrated, and the crude aldehyde was dried under high vacuum.

To a vigorously stirred solution of this crude product in acetone (50 mL) the Jones reagent (12 mL) was added dropwise at room temperature. After 1 h, 2-propanol (20 mL) was added, the resulting mixture was stirred for 30 min, and concentrated to ca. 20 mL. Water (50 mL) and AcOEt (100 mL) were added, the layers were separated, and the aqueous one washed with AcOEt (2 × 50 mL). Combined organic solutions were dried, concentrated, and the crude acid was dried under high vacuum.

To a solution of this crude material in DCM/pyridine (50 mL/10 mL) triphenylphosphine (1.6 g, 1.2 equiv in relation to iododerivative), tetrabromomethane (2 g, 1.2 equiv), and *N*,*O*-dimethylhydroxylamine hydrochloride (0.75 g, 1.5 equiv) were added, and the mixture was vigorously stirred at rt. for 24 h. Silica gel (30 g, 230-400 mesh) was added, the solvent was evaporated, and the residue was purified by column chromatography (100% hexanes to 100% AcOEt) to afford **15** or **16** respectively as colorless syrup.

(2*R*,3*S*,4*R*)-2,3,4-Tris(benzyloxy)-*N*-methoxy-*N*-methylhex-5-enamide (**15**):

Yield: 67% (1.59 g). HRMS: found: *m*/*z* = 498.2250; calcd. for $C_{29}H_{33}NO_5Na$ ($[M + Na]^+$): 498.2256. $[\alpha]_D^{23}$ = +29.0. R_f = 0.4 (hexanes/AcOEt 3:1). ^{1}H NMR (500 MHz) δ: 7.25 (m, arom.), 5.77 (m, 1H, H-5), 5.22 (m, 2H, H-6, H-6'), 4.74 (m, 3H, 3 × OC$\underline{H}_2$Ph), 4.54 (d, 1H, *J* = 11.6 Hz, OC$\underline{H}_2$Ph), 4.42 (m, 2H, OC$\underline{H}_2$Ph, H-2), 4.35 (d, 1H, *J* = 11.6 Hz, OC$\underline{H}_2$Ph), 4.13 (m, 1H, H-4), 3.96 (dd, 1H, *J* = 6.3, 4.5 Hz, H-3), 3.39 (s, 3H, OC$\underline{H}_3$), 3.01 ppm (s, 3H, C$\underline{H}_3$). ^{13}C NMR (125 MHz) δ: 170.6 ($\underline{C}$=O), 138.5, 137.6 (3 × quat. benzyl), 135.1 (C-5), 128.6-127.4 (arom.), 119.1 (C-6), 81.5 (C-4), 80.6 (C-3), 76.4 (C-2), 74.6, 72.5, 70.9 (3 × O$\underline{C}H_2$Ph), 60.9 (O$\underline{C}H_3$), 32.2 ppm ($\underline{C}H_3$).

(2*S*,3*S*,4*R*)-2,3,4-Tris(benzyloxy)-*N*-methoxy-*N*-methylhex-5-enamide (**16**):

Yield: 68% (1.61 g). HRMS: found: *m*/*z* = 498.2264; calcd. for $C_{29}H_{33}NO_5Na$ ($[M + Na]^+$): 498.2256. $[\alpha]_D^{23}$ = -14.0. R_f = 0.4 (hexanes/AcOEt 3:1). Anal.: found: C – 73.22, H – 6.89, N – 2.82%; calcd. C – 73.24, H – 6.99, N – 2.95%. ^{1}H NMR (600 MHz) δ: 7.25 (m, arom.), 5.95 (ddd, 1H, *J* = 17.6, 10.1, 7.8 Hz, H-5), 5.37 (dd, 1H, *J* = 17.3, 1.2 Hz, H-6), 5.27 (~d, 1H, *J* = 10.4 Hz, H-6'), 4.84 (d, 1H, *J* = 8.5 Hz, H-2), 4.63 (d, 1H, *J* = 12.0 Hz, OC$\underline{H}_2$Ph), 4.58 (d, 1H, *J* = 10.8 Hz, OC$\underline{H}_2$Ph), 4.50 (d, 1H, *J* = 10.8 Hz, OC$\underline{H}_2$Ph), 4.41 (d, 1H, *J* = 11.4 Hz, OC$\underline{H}_2$Ph), 4.26 (m, 2H, 2 × OC$\underline{H}_2$Ph), 4.14 (dd, 1H, *J* = 7.5, 2.4 Hz, H-4), 3.84 (dd, 1H, *J* = 8.8, 2.2 Hz, H-3), 3.54 (s, 3H, OC$\underline{H}_3$), 3.13 ppm (s, 3H, C$\underline{H}_3$). ^{13}C NMR (150 MHz, $CDCl_3$) δ: 172.1 ($\underline{C}$=O), 138.6, 138.1, 137.5 (3 × quat. benzyl), 135.9 (C-5), 128.4-127.4 (arom.), 118.3 (C-6), 81.5 (C-3), 80.6 (C-4), 75.6 (O$\underline{C}H_2$Ph), 73.0 (C-2), 71.9, 70.5 (2 × O$\underline{C}H_2$Ph), 61.6 (O$\underline{C}H_3$), 32.0 ppm ($\underline{C}H_3$).

Synthesis of cyclohexenones via RCM

These reactions were performed under an argon atmosphere.

To a vigorously stirred and cooled to 0 °C solution of Weinreb amide **15** or **16** (0.95 g, 2 mmol) in dry THF (15 mL), vinylmagnesium bromide (1M/THF, 4 mL) was added dropwise (syringe pump, 30 min), the mixture was stirred for additional 30 min at 0 °C, and then was quenched with 5% aq. HCl (20 mL). The layers were separated and the aqueous one extracted with Et_2O (3 × 50 mL). Combined organic solutions were dried, concentrated, and the crude diene was dried under high vacuum.

To a solution of this crude product in dry toluene (10 mL), the Hoveyda–Grubbs II generation catalyst (65 mg, 5 mol % in relation to Weinreb amide) was added and the reaction mixture was stirred at 50 °C for 24 h. Then it was concentrated and the residue was purified by chromatography (100% hexanes to 100% AcOEt) to afford **17** (brown amorphous solid) or **18** (brown syrup).

(4*R*,5*S*,6*R*)-4,5,6-Tris(benzyloxy)cyclohex-2-en-1-one (**17**):

Yield: 61% (505 mg). HRMS: found: *m*/*z* = 437.1724; calcd. for $C_{27}H_{26}O_4Na$ ($[M + Na]^+$): 437.1729. $[\alpha]_D^{23}$ = -63.6. R_f = 0.4 (hexanes/AcOEt 4:1). Anal.: found: C – 78.43, H – 6.38%; calcd. C – 73.24, H – 6.32%. ^{1}H NMR (500 MHz) δ: 7.34 (m, arom.), 6.81 (dd, 1H, *J* = 10.4, 2.0 Hz, H-3), 6.04 (dd, 1H, *J* = 10.4, 2.4 Hz, H-2), 5.08 (d, 1H, *J* = 11.4 Hz, OC$\underline{H}_2$Ph), 4.96 (d, 1H, *J* = 10.9 Hz, OC$\underline{H}_2$Ph), 4.82 (m, 2H, 2 × OC$\underline{H}_2$Ph), 4.74 (m, 2H, 2 × OC$\underline{H}_2$Ph), 4.36 (~dt, 1H, *J* = 8.0, 2.2 Hz, H-4), 4.04 (d, 1H, *J* = 10.7 Hz, H-6), 3.97 ppm (dd, 1H, *J* = 10.7, 8.0 Hz, H-5). ^{13}C NMR (125 MHz) δ: 197.4 ($\underline{C}$=O), 148.0 (C-3), 138.2, 137.8, 137.6 (3 × quat. benzyl), 128.5-127.7 (arom., C-2), 84.7 (C-5), 83.8 (C-6), 79.0 (C-4), 75.7, 74.5, 73.6 ppm (3 × O$\underline{C}H_2$Ph).

(4*R*,5*S*,6*S*)-4,5,6-Tris(benzyloxy)cyclohex-2-en-1-one (**18**):

Yield: 75% (621 mg). HRMS: found: *m*/*z* = 437.1734; calcd. for $C_{27}H_{26}O_4Na$ ($[M + Na]^+$): 437.1729. $[\alpha]_D^{23}$ = -104.0. R_f = 0.4 (hexanes/AcOEt 4:1). ^{1}H NMR (600 MHz) δ: 7.34 (m, arom.), 6.77 (dd, 1H, *J* = 10.2, 3.2 Hz, H-3), 5.99 (d, 1H, *J* = 10.2 Hz, H-2), 4.81 (d, 1H, *J* = 12.2 Hz, OC$\underline{H}_2$Ph), 4.70 (m, 2H, 2 × OC$\underline{H}_2$Ph), 4.61 (m, 2H, 2 × OC$\underline{H}_2$Ph), 4.54 (d, 1H, *J* = 12.2 Hz, OC$\underline{H}_2$Ph), 4.42 (~s, 1H, H-4), 4.23 (~s, 1H, H-6), 3.96 ppm (dd, 1H, *J* =5.6, 2.4 Hz, H-5). ^{13}C NMR (150 MHz) δ: 196.1 ($\underline{C}$=O), 146.1 (C-3), 137.8, 137.7, 137.5 (3 × quat. benzyl), 128.5-127.7 (arom., C-2), 79.9 (C-5), 78.9 (C-6), 75.1 (C-4), 73.2, 72.7, 72.4 ppm (3 × O$\underline{C}H_2$Ph).

Deprotection of silyl ethers

To a stirred and cooled to 0 °C solution of silyl ether **21** or **22** (3.96 g, 10 mmol) in THF (100 mL), TBAF·3H_2O (3.8 g, 1.2 equiv) was added. The cooling bath was removed, the mixture was stirred at room temperature for 24 h, and concentrated to ca. 20 mL, water (50 mL). Methylene dichloride (50 mL) was added. The layers were separated and the aqueous one extracted with DCM (2 × 30 mL). Combined organic solutions were dried, concentrated, and the residue was purified by chromatography (100% hexanes to 100% AcOEt) to yield **19** or **23** as colorless oil.

(*S*)-1-((*R*)-2,2-Dimethyl-1,3-dioxolan-4-yl)prop-2-en-1-ol (**19**):

Yield: 96% (1.52 g). All data matched those already reported [1].

(*R*)-1-((*R*)-2,2-Dimethyl-1,3-dioxolan-4-yl)prop-2-en-1-ol (**23**):

Yield: 94% (1.49 g). All data matched those already reported [2].

Benzoylation followed by removal of isopropylidene group

To a solution of the allylic alcohol **19** or **23** (1.58 g, 10 mmol) in DCM (100 mL), under vigorous stirring and at room temperature, Et_3N (10 mL) and DMAP (60 mg, 5 mol %) were added. Then, benzoyl chloride (3 mL, 2.5 equiv) was added dropwise over a period of 10 min and the resulting mixture was stirred for 24 h. Water (50 mL) was added, the layers were separated, and the aqueous one was washed with DCM (2 × 30 mL). Combined organic solutions were dried, concentrated, and the crude product was dried under high vacuum. To a solution of this crude material in MeOH (50 mL) 5% aq. HCl (5 mL) was added with vigorous stirring at rt, the resulting mixture was stirred at room temperature for 24 h, and concentrated to ca. 20 mL. The residue was partitioned between water (50 mL) and DCM (50 mL), the layers were separated, and the aqueous one was extracted with with DCM (2 × 30 mL). The combined organic solutions were dried, concentrated, and the residue was purified by chromatography (100% hexanes to 100% AcOEt) to afford **20** or **24** as yellow syrup.

(3*S*,4*R*)-4,5-Dihydroxypent-1-en-3-yl benzoate (**20**):

Yield: 55% (1.22 g). HRMS: found: *m*/*z* = 245.0791; calcd. for $C_{12}H_{14}O_4Na$ ($[M + Na]^+$): 245.0790. $[\alpha]_D^{23}$ = -58.9. R_f = 0.1 (hexanes/AcOEt 3:1). Anal.: found: C – 64.83, H – 6.24%; calcd. C – 64.85, H – 6.35%. ^{1}H NMR (600 MHz) δ: 8.07, 7.59, 7.46 (3 × m, arom.), 6.04 (ddd, 1H, *J* = 17.2, 10.6, 6.4 Hz, H-2), 5.54 (ddd, 1H, *J* = 6.3, 2.4, 1.2 Hz, H-3), 5.47 (~dt, 1H, *J* = 17.3, 1.3 Hz, H-1), 5.38 (~dt, 1H, *J* = 10.6, 1.2 Hz, H-1'), 3.91 (dd, 1H, *J* = 9.0, 5.8 Hz, H-4), 3.78 (dd, 1H, *J* = 11.7, 3.0 Hz, H-5), 3.69 ppm (dd, 1H, *J* = 11.8, 5.9 Hz, H-5'). ^{13}C NMR (150 MHz) δ: 166.0 (C=O), 133.4 (arom.), 132.8 (C-2), 129.7-128.5 (arom.), 119.3 (C-1), 75.3 (C-3), 73.0 (C-4), 62.6 ppm (C-5).

(3*R*,4*R*)-4,5-Dihydroxypent-1-en-3-yl benzoate (**24**):

Yield: 58% (1.29 g). HRMS: found: *m*/*z* = 245.0787; calcd. for $C_{12}H_{14}O_4Na$ ($[M + Na]^+$): 245.0790. $[\alpha]_D^{23}$ = +52.8. R_f = 0.1 (hexanes/AcOEt 3:1). Anal.: found: C – 64.90, H – 6.37%; calcd. C – 64.85, H – 6.35%. ^{1}H NMR (600 MHz) δ: 8.07, 7.58, 7.45 (3 × m, arom.), 5.97 (ddd, 1H, *J* = 17.1, 10.6, 6.4 Hz, H-2), 5.54 (m, 1H, H-3), 5.49 (~dt, 1H, *J* = 17.3, 1.2 Hz, H-1), 5.36 (~dt, 1H, *J* = 10.6, 1.2 Hz, H-1'), 3.94 (~td, 1H, *J* = 6.0, 4.4 Hz, H-4), 3.73 (dd, 1H, *J* = 11.6, 4.3 Hz, H-5), 3.69 ppm (dd, 1H, *J* = 11.6, 6.2 Hz, H-5'). ^{13}C NMR (150 MHz) δ: 166.1 (C=O), 133.4 (arom.), 132.6 (C-2), 129.7-128.5 (arom.), 119.3 (C-1), 75.6 (C-3), 73.3 (C-4), 63.0 ppm (C-5).

1. Schneider, C.; Kazmaier, U. *Synthesis* **1998**, 1314-1320.

2. Park, J. K.; McQuade, D. T. *Angew. Chem., Int. Ed.* **2012**, *51*, 2717-2721.

Diol cleavage with $NaIO_4$

To a stirred solution of diol **20** or **24** (220 mg, 1 mmol) in DCM (5 mL), saturated aq. $NaHCO_3$ (0.3 mL) was added followed by $NaIO_4$ (1 g, 4.7 equiv) added in few portions over a period of 5 min. After 24 h, $MgSO_4$ (200 mg) was added, stirring was continued for 15 min. and then the mixture was filtered through a pad of Celite which was then washed with DCM (30 mL). Solvent was evaporated and the crude aldehyde was dried under high vacuum. The product (aldehyde (*S*)-**10** or (*R*)-**10**, respectively) was used without further purification.

1,4-Addition of vinyl-MgBr/aldol reaction

This transformation was performed under an argon atmosphere.

To a stirred and cooled to −45 °C suspension of cyclohexanone **17** (207 mg, 0.5 mmol) and CuBr·Me_2S (50 mg, 0.5 equiv) in THF (2.5 mL), vinylmagnesium bromide (1 mL, 1 M in THF) was added via a syringe pump (5 min). After another 10 min, a solution of freshly prepared aldehyde (*S*)-**10** or (*R*)-**10** (from 1 mmol of diol **20** or **24**, respectively) in dry THF (1 mL) was added via a syringe pump (5 min). Once the addition was completed, the mixture was allowed to warm up to −20 °C (10 min) and the reaction was quenched with saturated aq. NH_4Cl (20 mL). Ethyl acetate (2 × 50 mL) was added, the layers were separated, the organic solution was dried and concentrated, and the crude products were isolated by chromatography (100% hexanes to 100% AcOEt) to yield diene **25** or **26** (dr = 10:1) as colorless syrup. These compounds are unstable and should be used immediately or stored at low temperatures.

Diene **25**:

Yield: 54% (170 mg). HRMS: found: *m/z* = 655.2673; calcd. for $C_{40}H_{40}O_7Na$ ($[M + Na]^+$): 655.2672. $[\alpha]_D^{23}$ = +25.4. R_f = 0.4 (hexanes/AcOEt 3:1). ^{1}H NMR (600 MHz) δ: 8.09 (arom.), 7.36 (arom.), 5.87 (m, 1H, H-9), 5.80 (m, 1H, H-8), 5.54 (m, 1H, H-11), 5.36 (m, 3H, H-12, H-12’, H-10), 5.27 (~d, 1H, *J* = 10.5 Hz, H-10), 4.91 (d, 1H, *J* = 10.8 Hz, OC*H*<u>2</u>Ph), 4.78 (d, 1H, *J* = 10.8 Hz, OC<u>*H*</u>$_2$Ph), 4.74 (d, 1H, *J* = 10.8 Hz, OC<u>*H*</u>$_2$Ph), 4.63 (m, 2H, 2 × OC<u>*H*</u>$_2$Ph), 4.34 (d, 1H, *J* = 11.6 Hz, OC<u>*H*</u>$_2$Ph), 4.10 (d, 1H, *J* = 8.7 Hz, H-6), 3.95 (ddd, 1H, *J* = 11.2, 6.2, 1.3 Hz, H-7), 3.66 (m, 2H, H-5, H-4), 3.55 (d, 1H, *J* = 11.3 Hz, O<u>*H*</u>), 2.74 (~dt, 1H, *J* = 12.3, 9.4 Hz, H-3), 2.49 ppm (~d, 1H, *J* = 12.5 Hz, H-2). ^{13}C NMR (150 MHz) δ: 208.1 (C-1), 166.1 (Ph<u>C</u>O-), 138.2, 138.0, 137.2 (3 × quat. benzyl), 136.0 (C-11), 133.8 (C-9), 133.1-127.7 (arom.), 121.1 (C-12), 119.5 (C-10), 85.5, 85.4, 82.1 (C-4, C-5, C-6), 78.2 (C-8), 75.8, 75.1, 73.0 (3 × quat. benzyl), 72.2 (C-7), 49.7 (C-2), 47.2 ppm (C-3).

Diene **26**:

Yield: 48% (152 mg). HRMS: found: *m/z* = 655.2661; calcd. for $C_{40}H_{40}O_7Na$ ($[M + Na]^+$): 655.2672. R_f = 0.5 (hexanes/AcOEt 3:1). ^{1}H NMR (600 MHz) δ: 8.08 (arom.), 7.39 (arom.), 6.12 (ddd, 1H, *J* = 17.1, 10.6, 6.1 Hz, H-9), 5.81 (m, 1H, H-8), 5.48 (m, 2H, H-10, H-11), 5.36 (m, 3H, H-10’, H-12, H-12’), 4.95 (m, 2H, 2 × OC<u>*H*</u>$_2$Ph), 4.74 (m, 2H, 2 × OC<u>*H*</u>$_2$Ph), 4.57 (m, 2H, 2 × OC<u>*H*</u>$_2$Ph), 4.15 (d, 1H, *J* = 9.5 Hz, H-6), 3.76 (m, 1H, H-7), 3.67 (m, 1H, H-5), 3.58 (dd, 1H, *J* = 10.0, 8.9 Hz, H-4), 3.52 (d, 1H, *J* = 12.0 Hz, H-), 2.72 (m, 1H, H-3), 2.38 ppm (d, 1H, *J* = 12.5 Hz,

H-2). ^{13}C NMR (150 MHz) δ: 208.9 (C-1), 166.8 (Ph<u>C</u>O-), 138.2, 138.0, 137.5 (3 × quat. benzyl), 136.7 (C-11), 134.3 (C-9), 133.5-127.6 (arom.), 120.8 (C-12), 118.5 (C-10), 85.7, 85.2, 82.4 (C-4, C-5, C-6), 75.9 (quat. benzyl), 75.4 (C-8), 75.1, 72.6 (2 × quat. benzyl), 71.3 (C-7), 47.7 (C-2), 46.7 ppm (C-3).

RCM followed by *syn*-dihydroxylation

This reaction was carried out as described in [3][4].

RCM reaction was performed under an argon atmosphere.

To a stirred solution of **25** or **26** (105 mg, 0.17 mmol) in dry toluene (0.8 mL), Hoveyda-Grubbs II generation catalyst (11 mg, 5 mol %) was added and the mixture was kept at 50 °C for 2 h. Then, solvent was evaporated, the residue was dissolved in AcOEt (0.5 mL) and MeCN (0.5 mL), and the resulting mixture was cooled to 0 °C. Simultaneously, in a separate vial, $NaIO_4$ (110 mg, 3.1 equiv) and $CeCl_3 \cdot 7H_2O$ (12 mg, 20 mol %) were suspended in water (0.1 mL) and the mixture was gently heated at 50 °C until it turned yellow (ca. 1 min); then, MeCN (0.2 mL) was added; the yellow suspension was cooled to 0 °C and added in one portion to the solution of the RCM product and the mixture was vigorously stirred for 20 min at 0 °C. Then, pulverized $MgSO_4$ (250 mg) and Na_2SO_3 (500 mg) were added and stirring was continued for 30 min. After this time, the mixture was filtered through a pad of Celite, which was then repeatedly washed with ethyl acetate. Solvent was evaporated and the residue was purified by chromatography (preparative TLC, 1 mm, DCM: MeOH 15:1) to yield either **27** and **28** (dr = 8:1) or **29** as a single isomer (all compounds obtained as white amorphous solids).

Decalin **27**:

Yield: 50% (54 mg). HRMS: found: *m*/*z* = 661.2418; calcd. for $C_{38}H_{38}O_9Na$ ($[M + Na]^+$): 661.2414. Anal. found: C – 71.38, H – 6.02%; calcd. C – 71.46, H – 6.00%. $[\alpha]_D^{23}$ = +51.2. R_f = 0.6 (DCM: methanol 10:1). ^{1}H NMR (600 MHz) δ: 8.08, 7.39 (arom.), 5.32 (~t, 1H, *J* = 9.6 Hz, H-8), 4.97 (m, 2H, 2 × OC<u>*H*</u>$_2$Ph), 4.88 (d, 1H, *J* = 11.4 Hz, OC<u>*H*</u>$_2$Ph), 4.79 (m, 2H, 2 × OC<u>*H*</u>$_2$Ph), 4.54 (d, 1H, *J* = 11.4 Hz, OC<u>*H*</u>$_2$Ph), 4.26 (d, 1H, *J* = 2.3 Hz, H-10), 4.18 (dd, 1H, *J* = 9.8, 1.1 Hz, H-4), 4.14 (~td, 1H, *J* = 9.7, 3.4 Hz, H-7), 4.07 (dd, 1H, *J* = 10.5, 9.2 Hz, H-2), 3.71 (~t, 1H, *J* = 9.5 Hz, H-3), 3.58 (m, 1H, H-9), 3.29 (~d, 1H, *J* = 4.7 Hz, O<u>*H*</u>), 3.13 (d, 1H, *J* = 3.4 Hz, O<u>*H*</u>), 2.86 (dd, 1H, *J* = 13.2, 10.2 Hz, H-), 2.52 (d, 1H, *J* = 1.8 Hz, H-6), 1.50 ppm (~t, 1H, *J* = 12.1 Hz, H-1). ^{13}C NMR (150 MHz) δ: 206.9 (C-5), 168.3 (Ph<u>C</u>O$_2$), 138.2, 138.0, 137.3 (3 × quat. benzyl), 86.2 (C-3), 85.9 (C-4), 77.2 (C-8), 76.9 (C-2), 75.9, 75.5, 73.6 (3 × O<u>C</u>H$_2$Ph), 73.2 (C-9), 69.2 (C-7), 67.9 (C-10), 47.4 (C-6), 41.4 ppm (C-1).

Decalin **28**:

Yield: 6% (7 mg). HRMS: found: *m*/*z* = 661.2402; calcd. for $C_{38}H_{38}O_9Na$ ($[M + Na]^+$): 661.2414. Anal. found: C – 71.23, H – 5.98%; calcd. C – 71.46, H – 6.00%. $[\alpha]_D^{23}$ = +61.4. R_f = 0.7 (DCM:

---

3. Malik, M.; Witkowski, G.; Ceborska, M.; Jarosz, S. *Org. Lett.* **2013**, 15, 6214-6217.
4. Malik, M.; Ceborska, M.; Witkowski, G.; Jarosz, S. *Tetrahedron: Asymmetry* **2015**, 26, 29-34.

methanol 10:1). ^{1}H NMR (600 MHz) δ: 8.13, 7.37 (arom.), 5.15 (d, 1H, *J* = 10.7 Hz, O*C̲*H$_2$Ph), 5.05 (m, 2H, O*C̲*H$_2$Ph, H-8), 4.92 (d, 1H, *J* = 11.3 Hz, O*C̲*H$_2$Ph), 4.78 (m, 2H, 2 × O*C̲*H$_2$Ph), 4.68 (~t, 1H, *J* = 10.0 Hz, H-7), 4.54 (d, 1H, *J* = 11.3 Hz, O*C̲*H$_2$Ph), 4.20 (~t, 1H, *J* = 2.5 Hz, H-9), 4.14 (m, 1H, H-4), 4.02 (dd, 1H, *J* = 10.4, 9.0 Hz, H-2), 3.85 (m, 1H, H-10), 3.81 (m, 1H, H-3), 2.29 (dd, 1H, *J* = 13.5, 9.8 Hz, H-6), 2.03 ppm (m, 1H, H-1). ^{13}C NMR (150 MHz) δ: 203.7 (C-5), 166.2 (Ph*C̲*O$_2$), 137.7, 137.1, 136.3 (3 × quat. benzyl), 86.0 (C-3), 85.8 (C-4), 83.5 (C-2), 75.9 (2 × O*C̲*H$_2$Ph), 74.1 (C-8), 73.8 (C-10), 73.6 (O*C̲*H$_2$Ph), 70.4 (C-9), 66.3 (C-7), 50.3 (C-6), 38.5 ppm (C-1).

Decalin **29**:

Yield: 53% (57 mg). HRMS: found: *m/z* = 661.2405; calcd. for $C_{38}H_{38}O_9Na$ ([M + Na]$^+$): 661.2414. Anal. found: C – 71.21, H – 6.17%; calcd. C – 71.46, H – 6.00%. $[\alpha]_D^{23}$ = +62.8. R_f = 0.4 (DCM: methanol 20:1). ^{1}H NMR (600 MHz) δ: 7.50 (m, arom.), 5.67 (m, 1H, H-8), 5.16 (d, 1H, *J* = 10.6 Hz, OC*H̲*$_2$Ph), 5.06 (d, 1H, *J* = 10.8 Hz, OC*H̲*$_2$Ph), 4.92 (m, 2H, OC*H̲*$_2$Ph), 4.78 (d, 1H, *J* = 10.4 Hz, OC*H̲*$_2$Ph), 4.61 (~d, 1H, *J* = 9.9 Hz, H-7), 4.52 (d, 1H, *J* = 11.4 Hz, OC*H̲*$_2$Ph), 4.22 (dd, 1H, *J* = 9.5, 1.0 Hz, H-4), 4.08 (dd, 1H, *J* = 10.5, 9.0 Hz, H-2), 4.05 (m, 1H, H-9), 3.97 (dd, 1H, *J* = 9.5, 3.0 Hz, H-10), 3.83 (~t, 1H, *J* = 9.2 Hz, H-3), 2.66 (dd, 1H, *J* = 13.4, 10.6 Hz, H-6), 1.95 ppm (~dt, 1H, *J* = 13.4, 10.1, H-1). ^{13}C NMR (150 MHz) δ: 205.2 (C-5), 165.2 (Ph*C̲*O$_2$), 137.8, 137.2, 136.3 (3 × quat. benzyl), 86.03 (C-3), 85.97 (C-4), 83.8 (C-2), 75.9, 75.8, 73.5 (3 × O*C̲*H$_2$Ph), 72.2 (C-8), 72.1 (C-10), 69.5 (C-9), 47.5 (C-6), 38.5 ppm (C-1).

Reduction of ketone with NaBH(OAc)$_3$

To a stirred solution of **27** or **29** (50 mg, 0.08 mmol) in MeCN/THF (0.4 mL/0.4 mL), AcOH was added (0.05 mL), followed by NaBH(OAc)$_3$ (85 mg, 5 equiv), and the mixture was stirred at room temperature for 24 h. Then, it was filtered through a pad of Celite, which was then repeatedly washed with ethyl acetate. Solvent was evaporated and the residue was purified by chromatography (preparative TLC, 1 mm, DCM: MeOH 10:1).

Decalin **30**:

Yield: 67% (34 mg, amorphous white solid). HRMS: found: *m/z* = 663.2566; calcd. for $C_{38}H_{40}O_9$ ([M + Na]$^+$): 663.2570. R_f = 0.3 (DCM: methanol 20:1). ^{1}H NMR (500 MHz) δ: 8.04, 7.36 (arom.), 5.27 (~t, 1H, *J* = 9.6 Hz, H-8), 4.89 (m, 3H, 3 × OC*H̲*$_2$Ph), 4.70 (m, 3H, 3 × OC*H̲*$_2$Ph), 4.42 (s, 1H, H-5), 4.22 (s, 1H, H-10), 3.96 (~t, 1H, *J* = 9.8 Hz, H-7), 3.89 (~t, 1H, *J* = 9.4 Hz, H-3), 3.65 (m, 1H, H-2), 3.60 (m, 1H, H-9), 3.44 (dd, 1H, *J* = 9.5, 2.8 Hz, H-4), 1.87 (m, 1H, H-1), 1.78 ppm (m, 1H, H-6). ^{13}C NMR (125 MHz) δ: 168.4 (Ph*C̲*O$_2$), 138.9, 138.6, 137.9 (3 × quat. benzyl), 83.3 (C-3), 82.9 (C-4), 79.5 (C-8), 78.3 (C-2), 75.7, 75.0 (2 × O*C̲*H$_2$Ph), 73.4 (C-9), 72.6 (O*C̲*H$_2$Ph), 69.7 (C-7), 68.4 (C-10), 65.0 (C-5), 39.0 (C-6), 38.5 ppm (C-1).

Decalin **31**:

Yield: 59% (30 mg, amorphous white solid). HRMS: found: *m/z* = 641.2741; calcd. for $C_{38}H_{41}O_9$ ([M + H]$^+$): 641.2751. $[\alpha]_D^{23}$ = +38.2. R_f = 0.3 (DCM: methanol 20:1). ^{1}H NMR (600 MHz) δ: 7.43 (m, arom.), 5.63 (m, 1H, H-8), 5.13 (d, 1H, *J* = 10.6 Hz, OC*H̲*$_2$Ph), 5.01 (d, 1H, *J* = 10.9 Hz, OC*H̲*$_2$Ph), 4.80 (d, 1H, *J* = 10.9 Hz, OC*H̲*$_2$Ph), 4.72 (m, 2H, 2 × OC*H̲*$_2$Ph), 4.63 (d, 1H, *J* = 11.5 Hz,

OC$\underline{H}_2$Ph), 4.48 (dd, 1H, *J* = 10.8, 2.8 Hz, H-7), 4.42 (m, 1H, H-5), 4.04 (~t, 1H, *J* = 3.5 Hz, H-9), 3.99 (~t, 1H, *J* = 9.3 Hz, H-3), 3.92 (dd, 1H, *J* = 10.0, 3.2 Hz, H-10), 3.66 (dd, 1H, *J* = 10.3, 9.3 Hz, H-2), 3.49 (dd, 1H, *J* = 9.4, 2.9 Hz, H-4), 2.36 (m, 1H, H-1), 1.58 ppm (m, 1H, H-6). ^{13}C NMR (150 MHz) δ: 166.2 (Ph$\underline{C}O_2$), 138.5, 137.7, 137.0 (3 × quat. benzyl), 85.7 (C-2), 83.13, 83.10 (C-3, C-4), 75.54, 75.50 (2 × O$\underline{C}H_2$Ph), 74.0 (C-8), 72.7 (C-10), 72.1 (O$\underline{C}H_2$Ph), 70.0 (C-9), 65.2 (C-7), 64.4 (C-5), 39.3 (C-6), 36.0 ppm (C-1).

Decalin **32**:

Yield: 15% (8 mg, colorless syrup). HRMS: found: *m*/*z* = 641.2749; calcd. for $C_{38}H_{41}O_9$ ([M + H]$^+$): 641.2751. $[\alpha]_D^{23}$ = +41.3. R_f = 0.25 (DCM: methanol 20:1). ^{1}H NMR (600 MHz) δ: 7.43 (m, arom.), 5.55 (m, 1H, H-8), 5.18 (s, 1H, O$\underline{H}$), 5.14 (d, 1H, *J* = 10.6 Hz, OC$\underline{H}_2$Ph), 5.00 (d, 1H, *J* = 11.0 Hz, OC$\underline{H}_2$Ph), 4.91 (m, 2H, 2 × OC$\underline{H}_2$Ph), 4.82 (d, 1H, *J* = 11.2 Hz, OC$\underline{H}_2$Ph), 4.75 (d, 1H, *J* = 10.6 Hz, OC$\underline{H}_2$Ph), 4.31 (dd, 1H, *J* = 9.8, 2.9 Hz, H-7), 4.03 (m, 1H, H-9), 3.92 (dd, 1H, *J* = 9.5, 3.2 Hz, H-10), 3.76 (m, 2H, H-5, H-2), 3.64 (m, 1H, H-3), 3.49 (~t, 1H, *J* = 9.2 Hz, H-4), 1.79 (m, 1H, H-1), 1.74 ppm (m, 1H, H-6). ^{13}C NMR (150 MHz) δ: 166.0 (Ph$\underline{C}O_2$), 138.2, 138.1, 136.5 (3 × quat. benzyl), 85.51, 85.46 (C-3, C-4), 84.6 (C-2), 76.8 (C-5), 75.78, 75.71, 75.5 (3 × O$\underline{C}H_2$Ph), 73.0 (C-8), 71.6 (C-10), 71.2 (C-7), 69.5 (C-9), 39.6 (C-6), 38.6 ppm (C-1).

Compound **15**, ^{1}H NMR, 500 MHz, $CDCl_3$

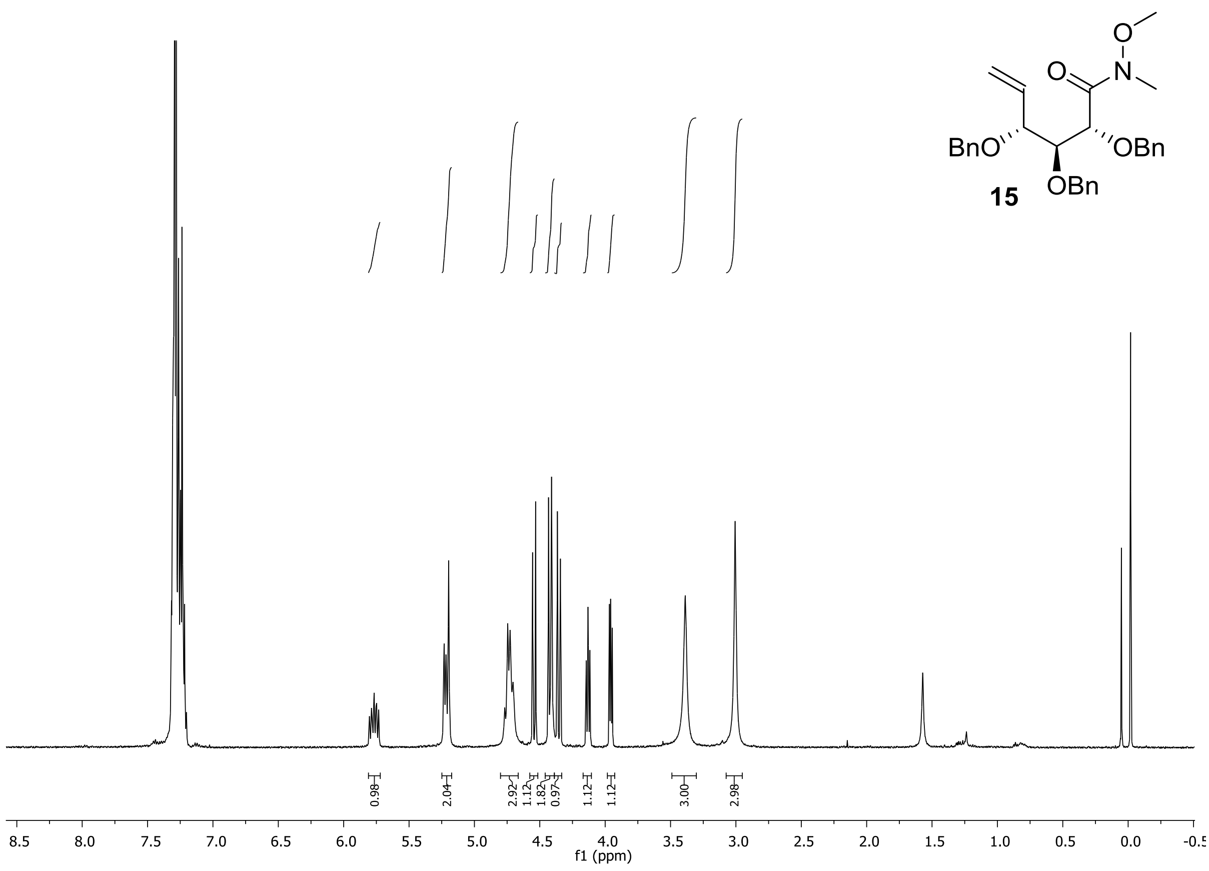

Compound **15**, ^{13}C NMR, 125 MHz, $CDCl_3$

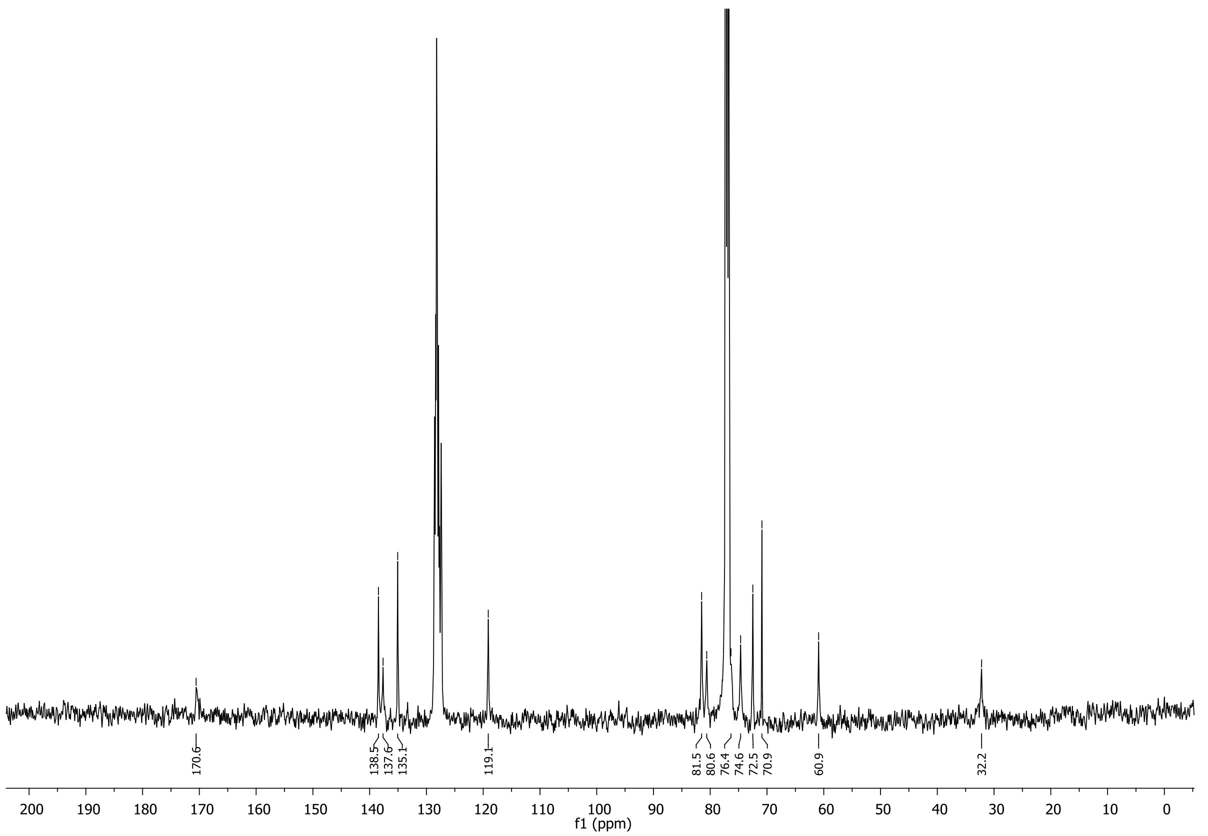

Compound **16**, ^{1}H NMR, 600 MHz, $CDCl_3$

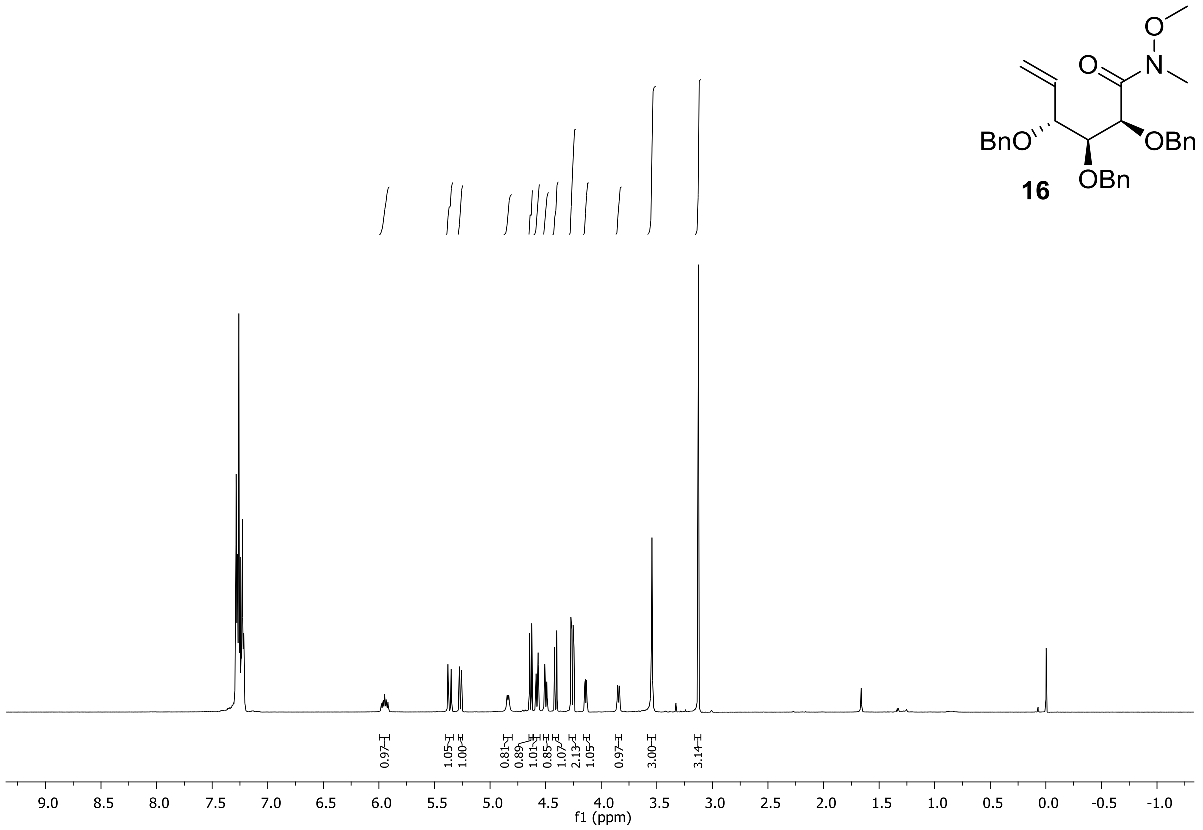

Compound **16**, ^{13}C NMR, 150 MHz, $CDCl_3$

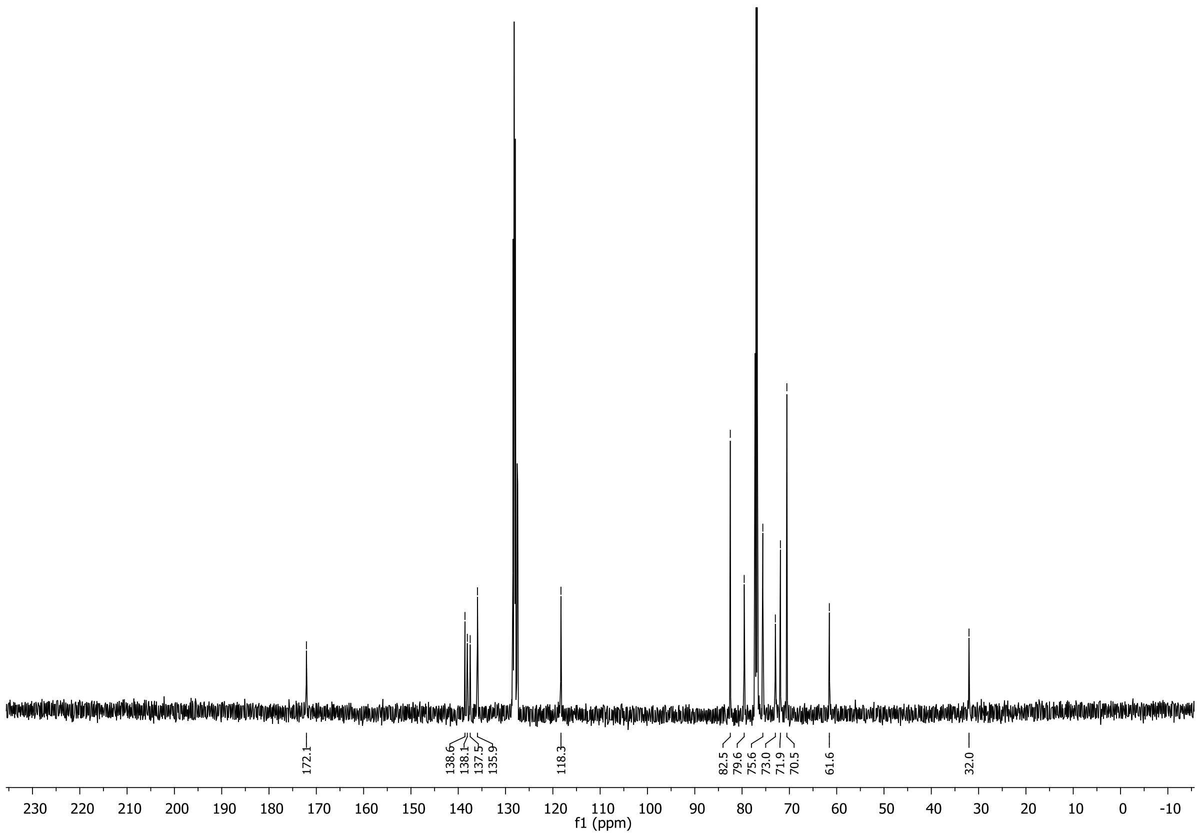

Compound **17**, ^{1}H NMR, 500 MHz, $CDCl_3$

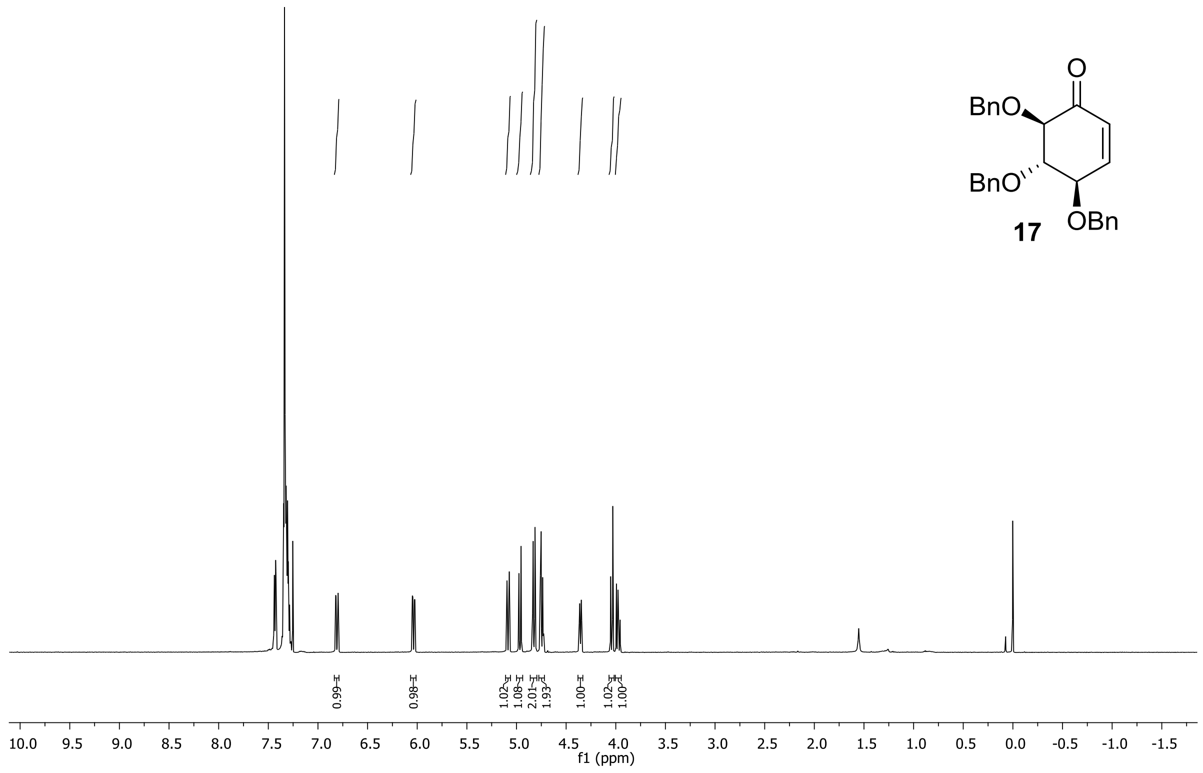

Compound **17**, ^{13}C NMR, 125 MHz, $CDCl_3$

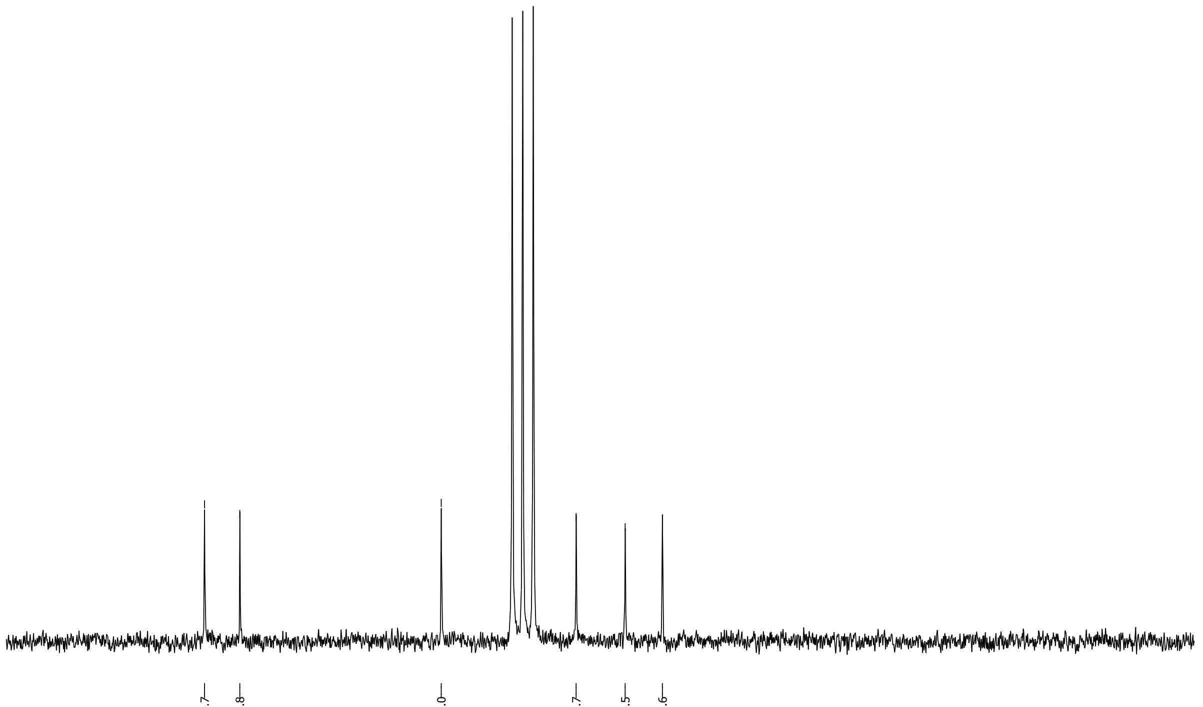

Compound **18**, ^{1}H NMR, 600 MHz, $CDCl_3$

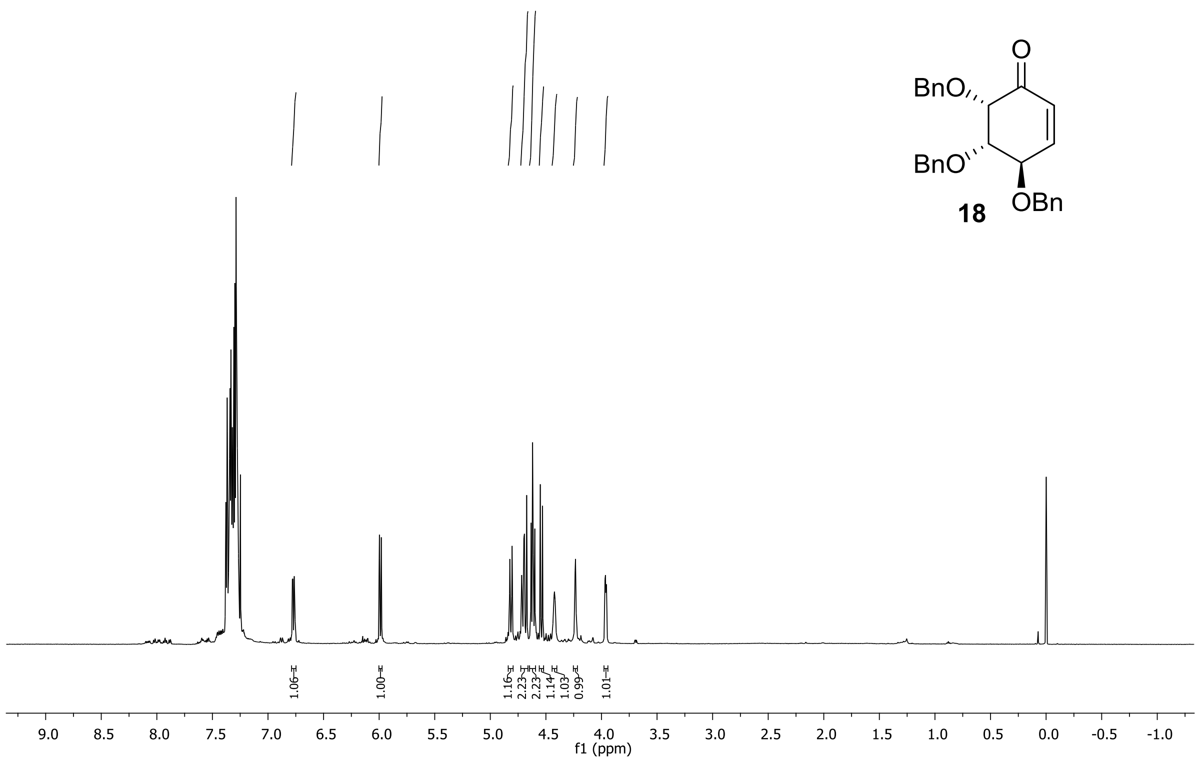

Compound **18**, ^{13}C NMR, 150 MHz, $CDCl_3$

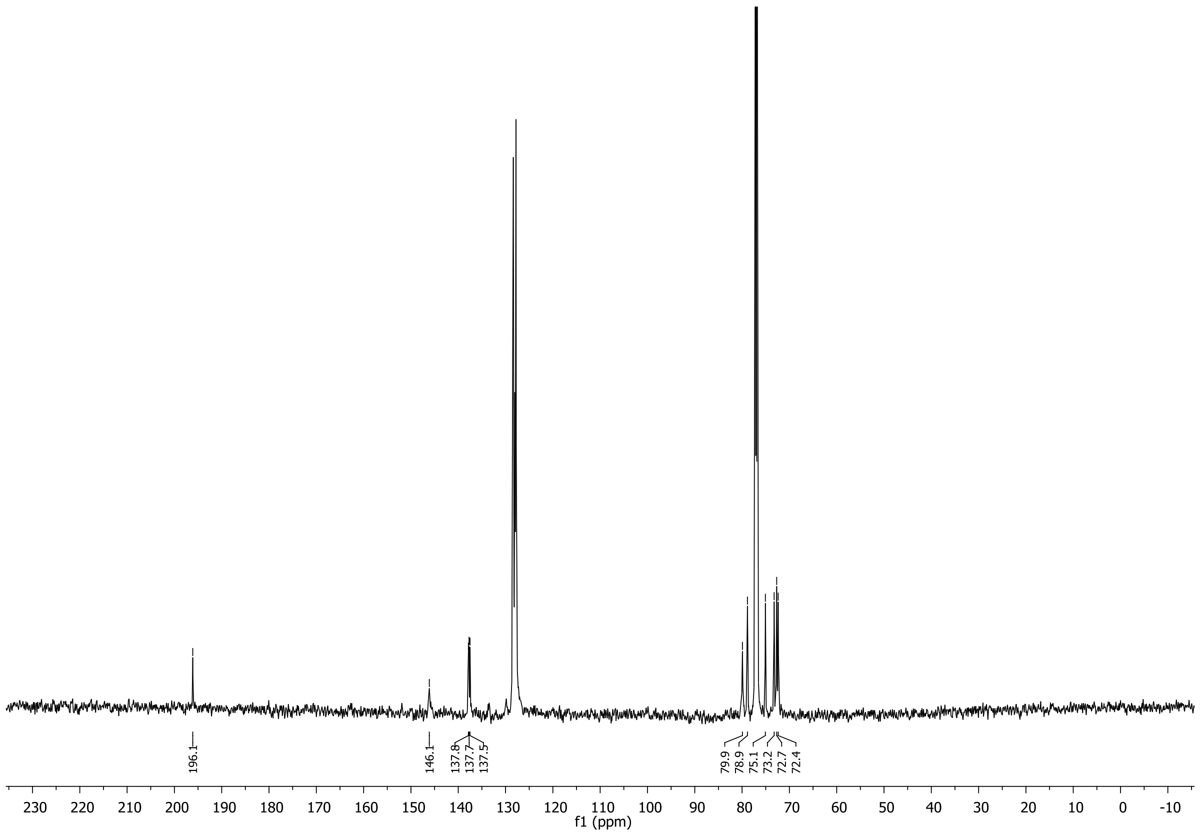

Compound **20**, ^{1}H NMR, 600 MHz, $CDCl_3$

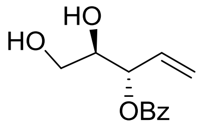

20

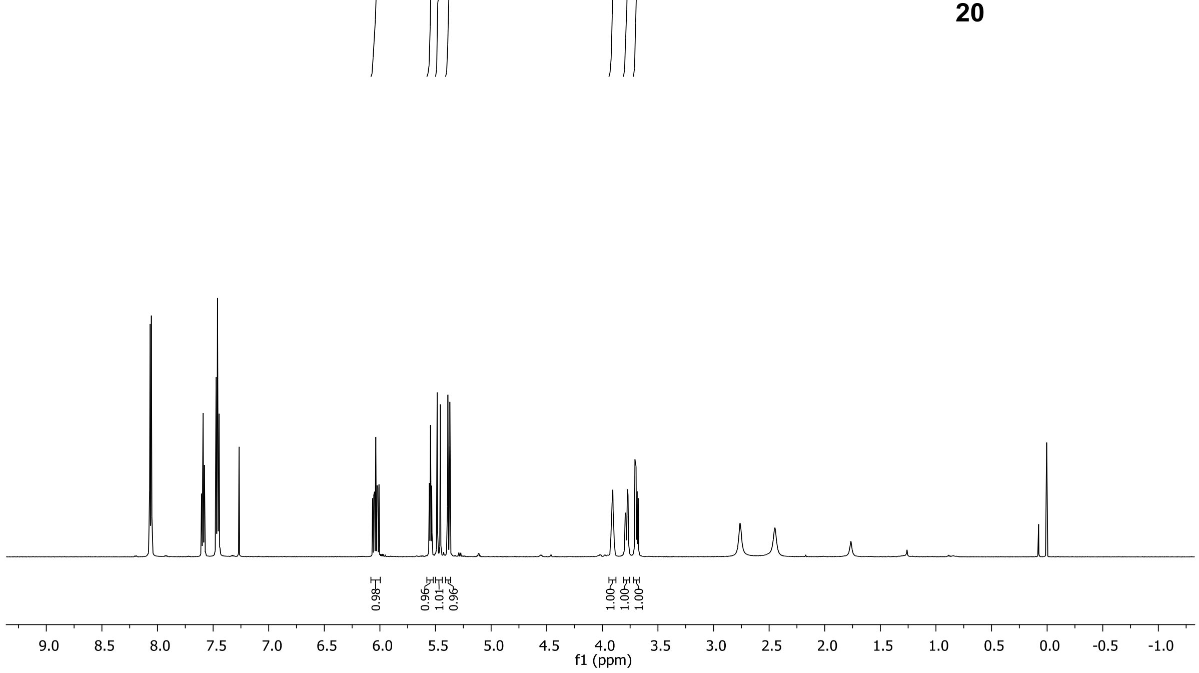

Compound **20**, ^{1}H NMR, 150 MHz, $CDCl_3$

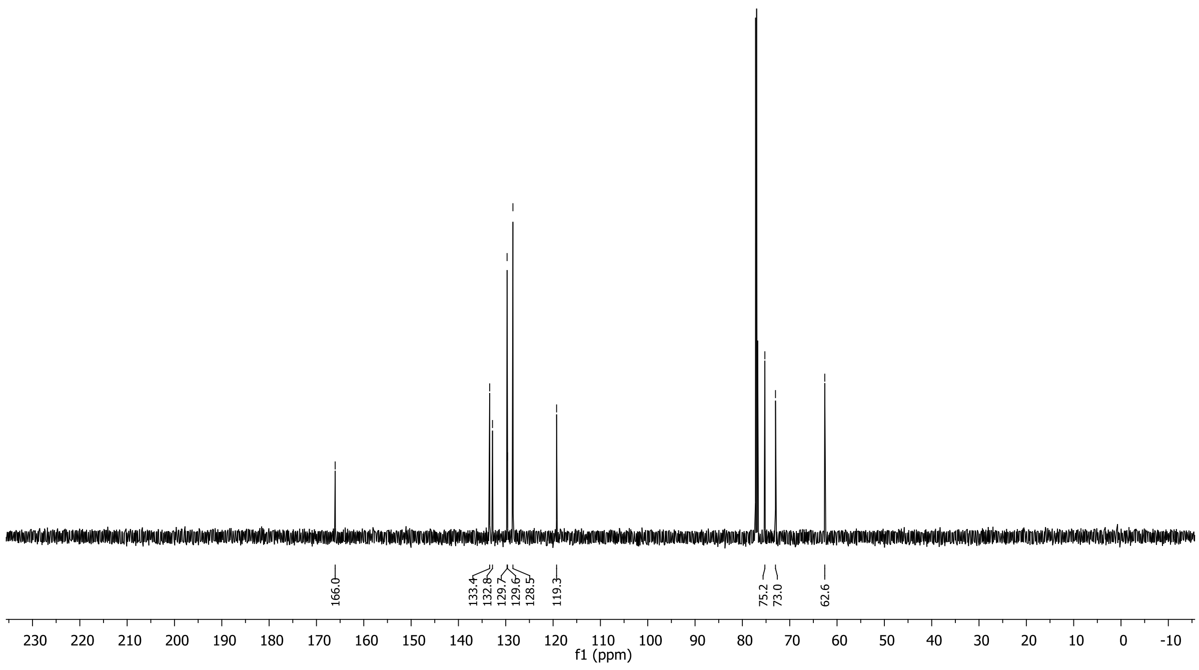

Compound **24**, ^{1}H NMR, 600 MHz, $CDCl_3$

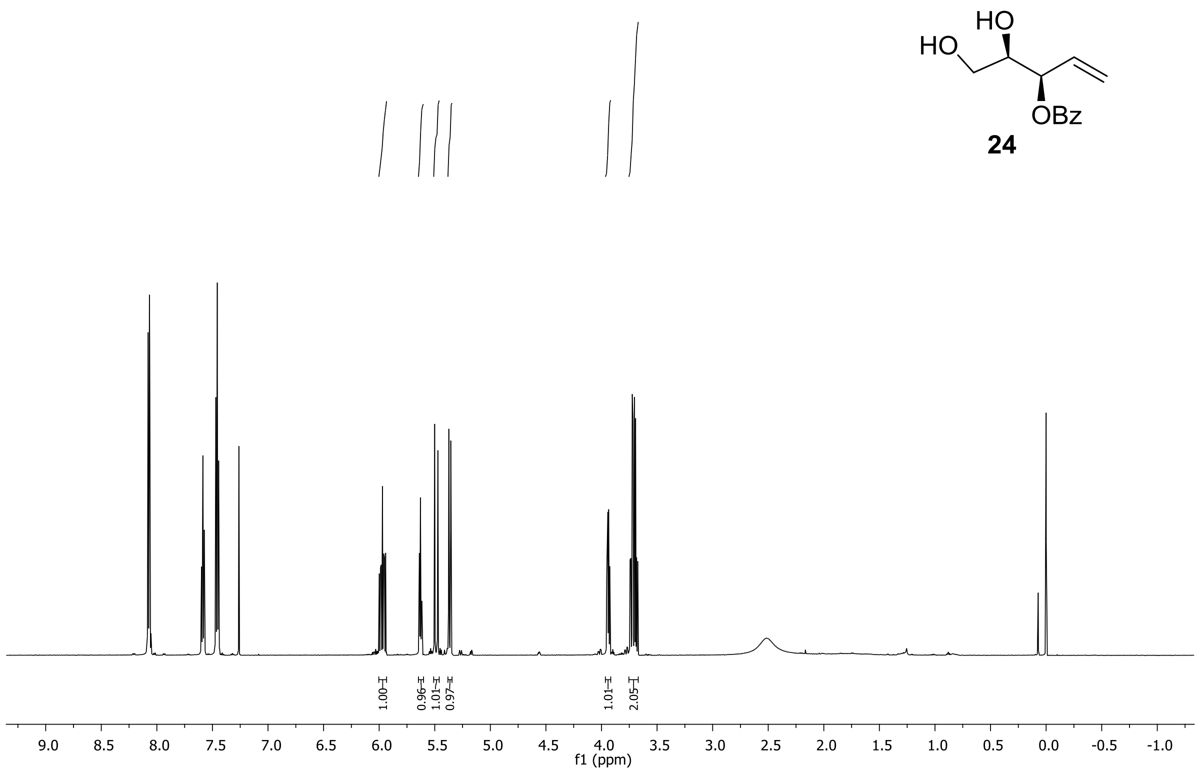

Compound **24**, ^{13}C NMR, 150 MHz, $CDCl_3$

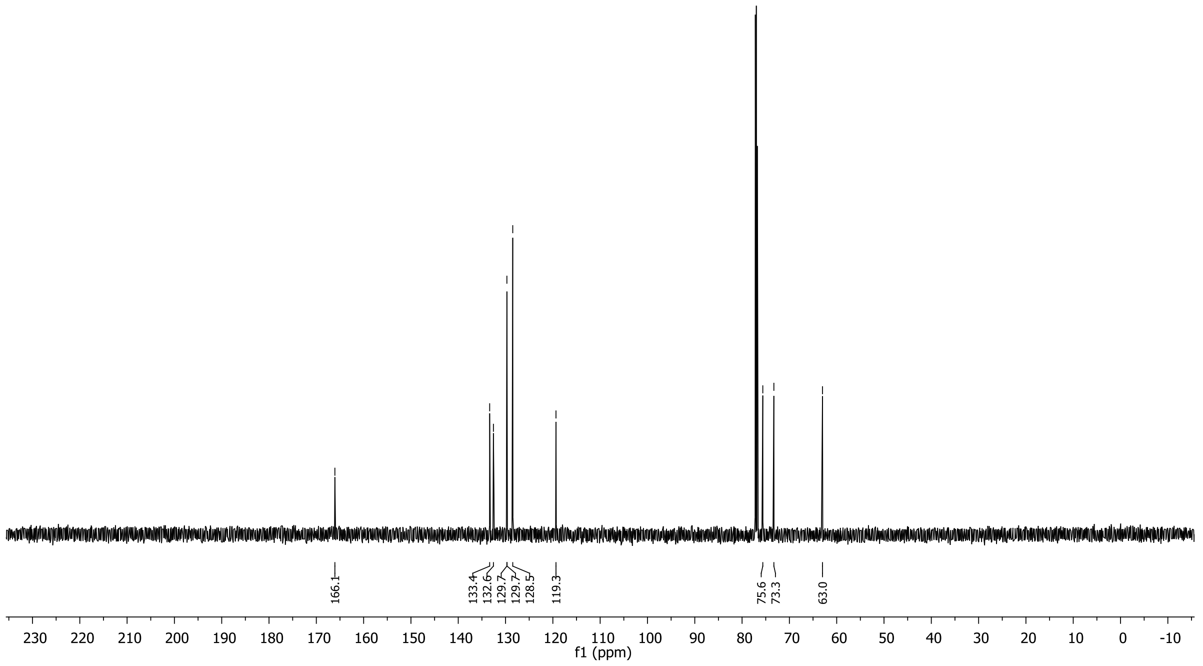

Compound **25**, ^{1}H NMR, 600 MHz, $CDCl_3$

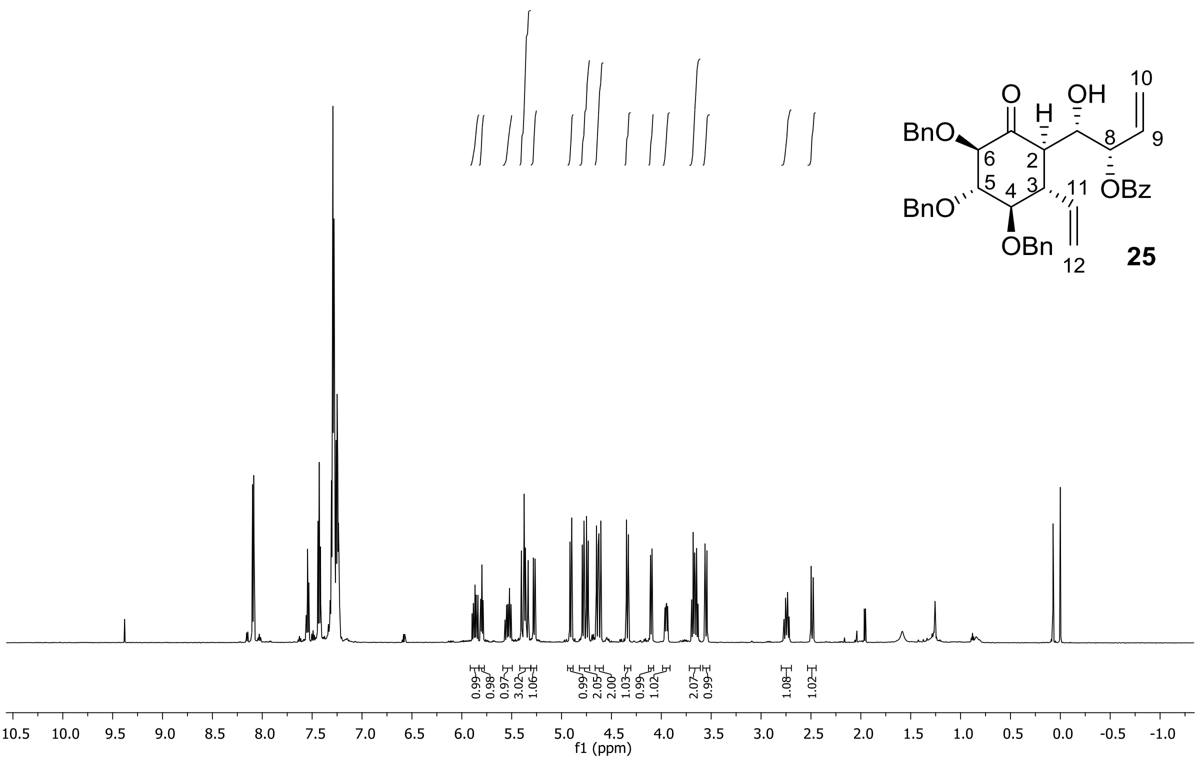

Compound **25**, ^{13}C NMR, 150 MHz, $CDCl_3$

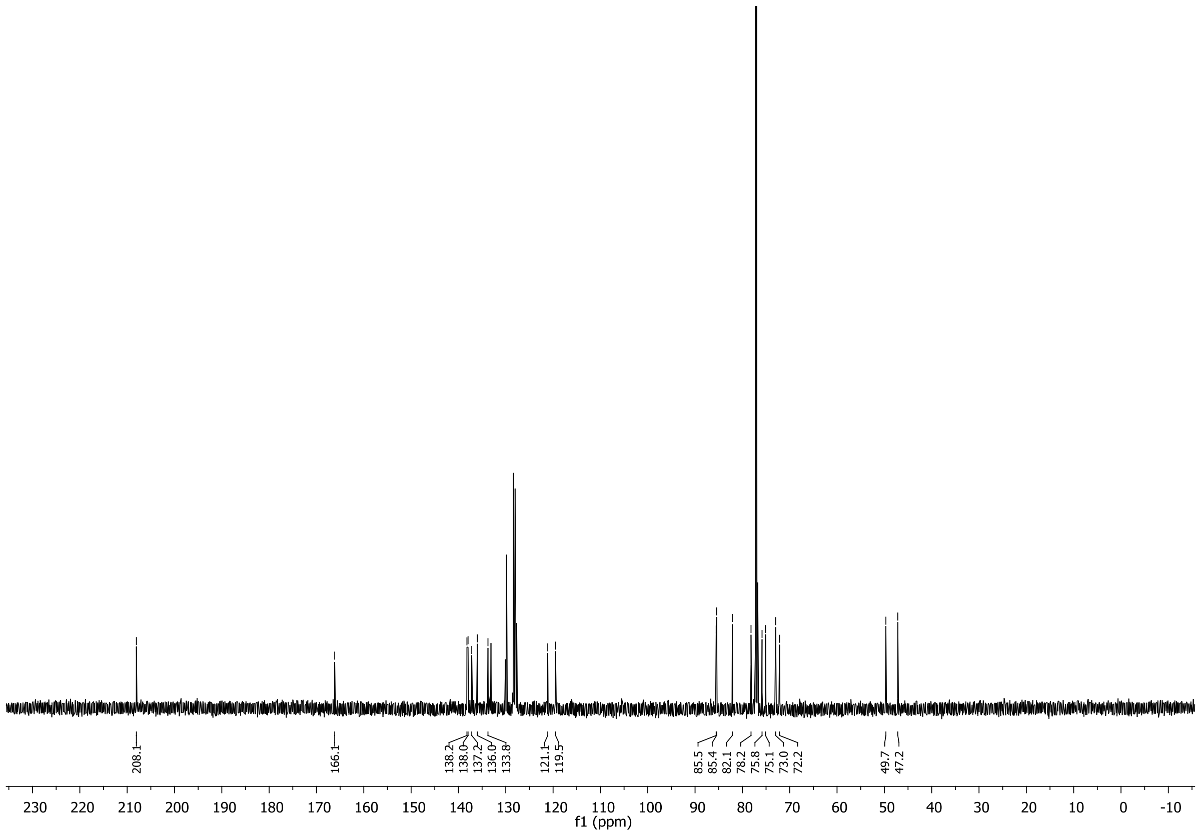

Compound **26**, ^{1}H NMR, 600 MHz, $CDCl_3$

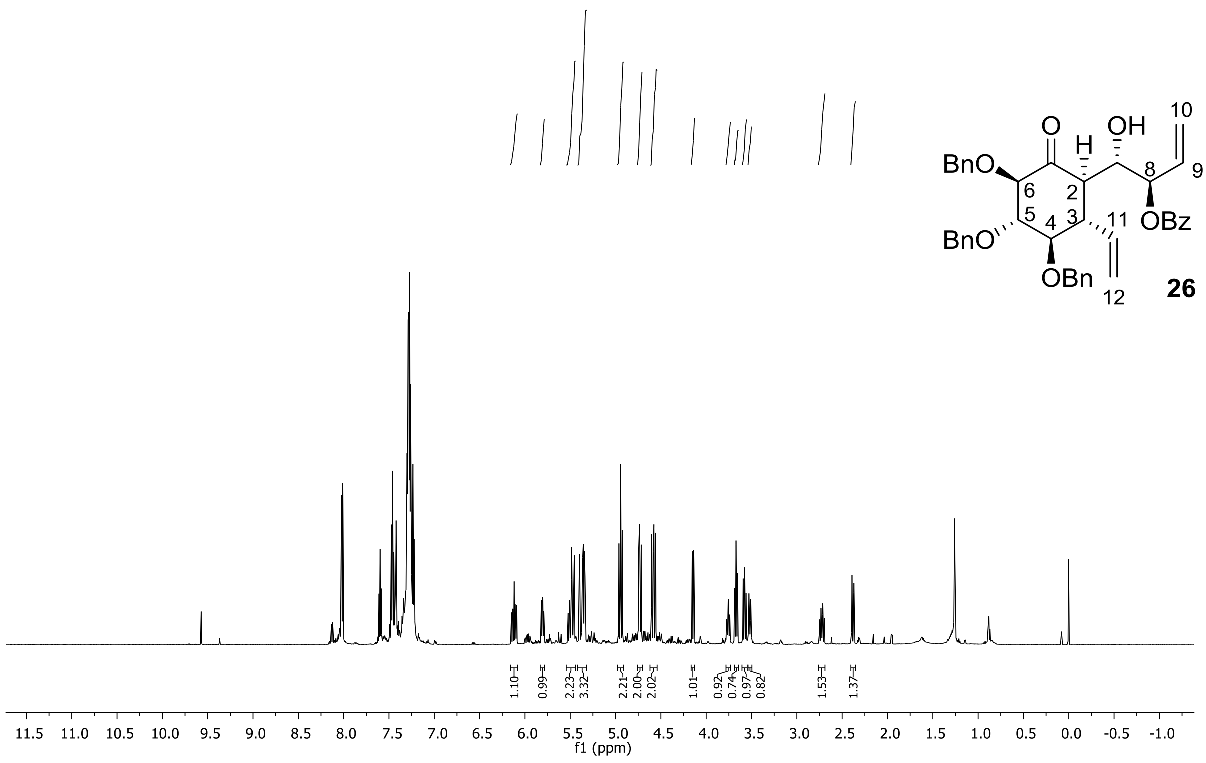

Compound **26**, ^{13}C NMR, 150 MHz, $CDCl_3$

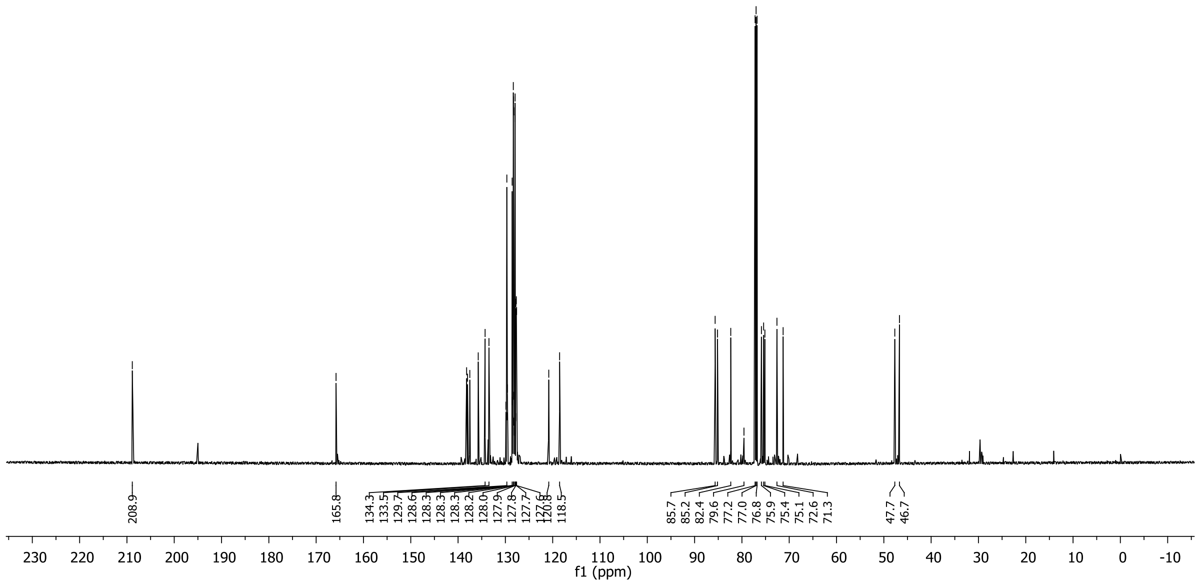

Compound **27**, ^{1}H NMR, 600 MHz, $CDCl_3$

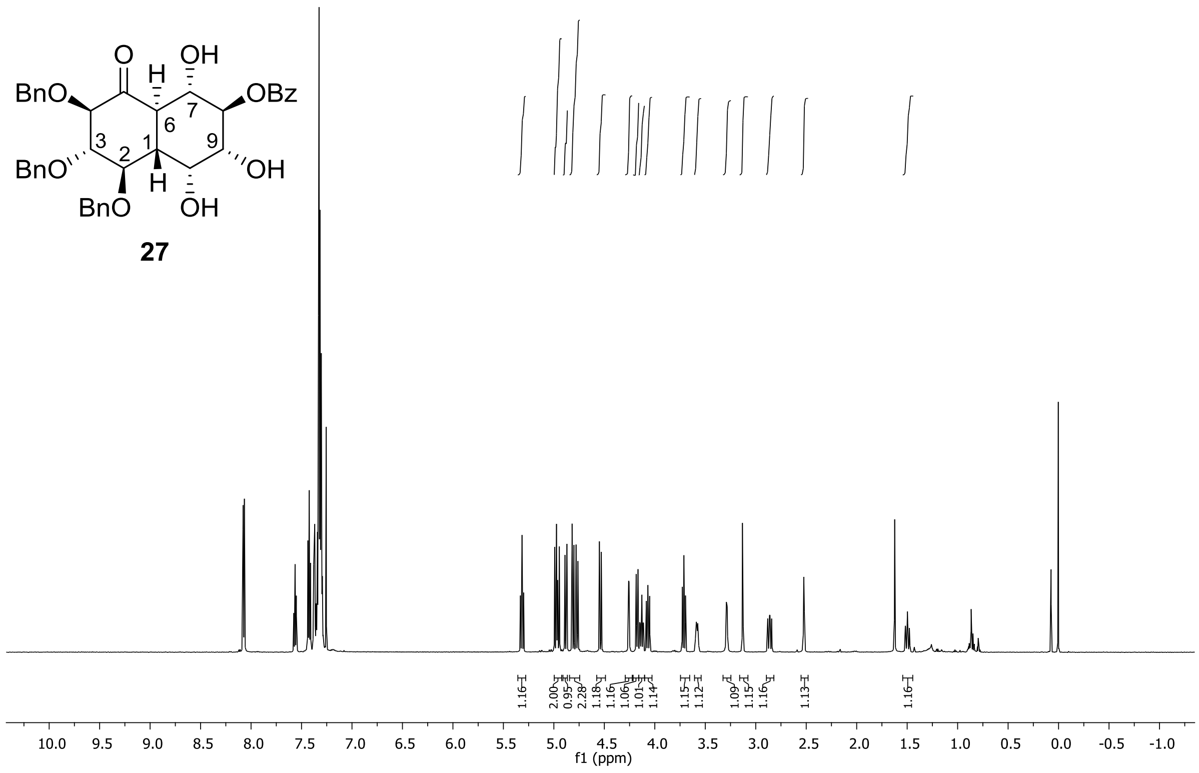

Compound **27**, ^{13}C NMR, 150 MHz, $CDCl_3$

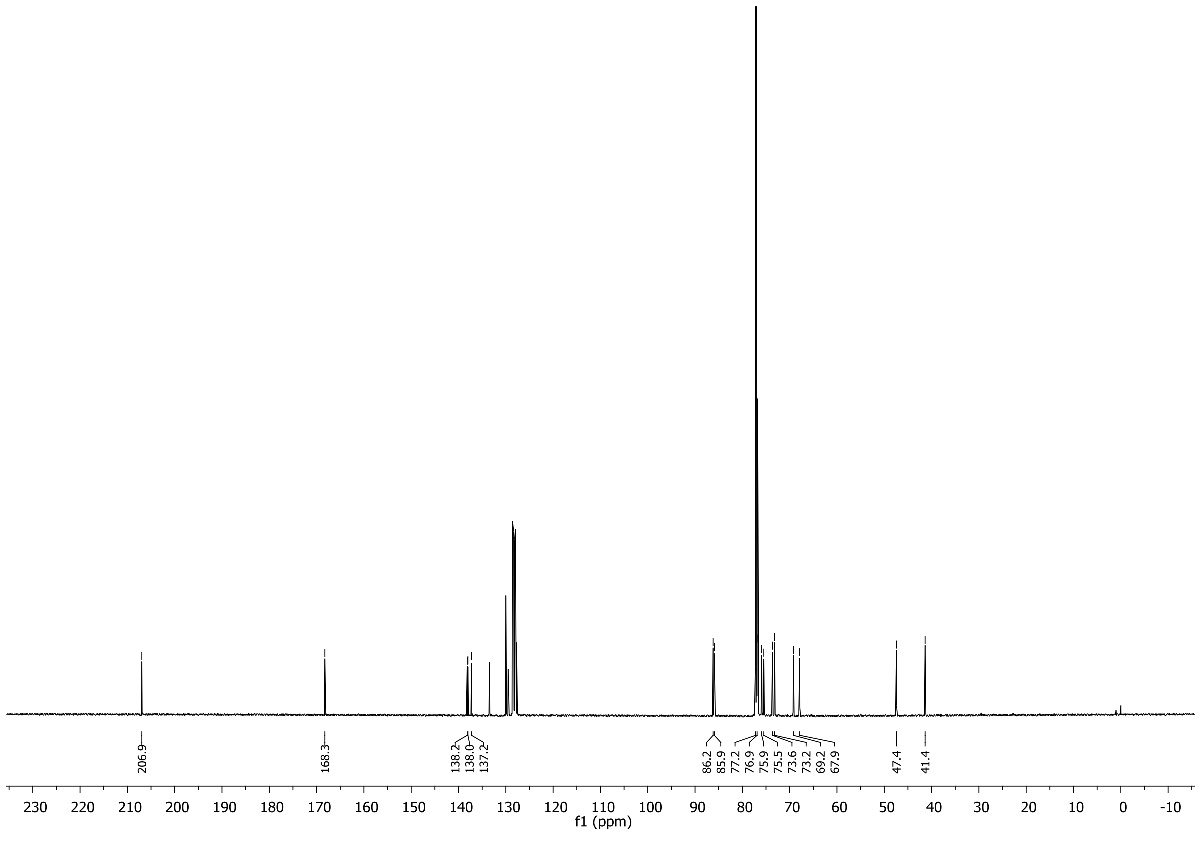

Compound **28**, ^{1}H NMR, 600 MHz, $CDCl_3$

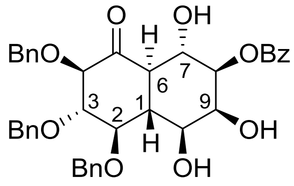

28

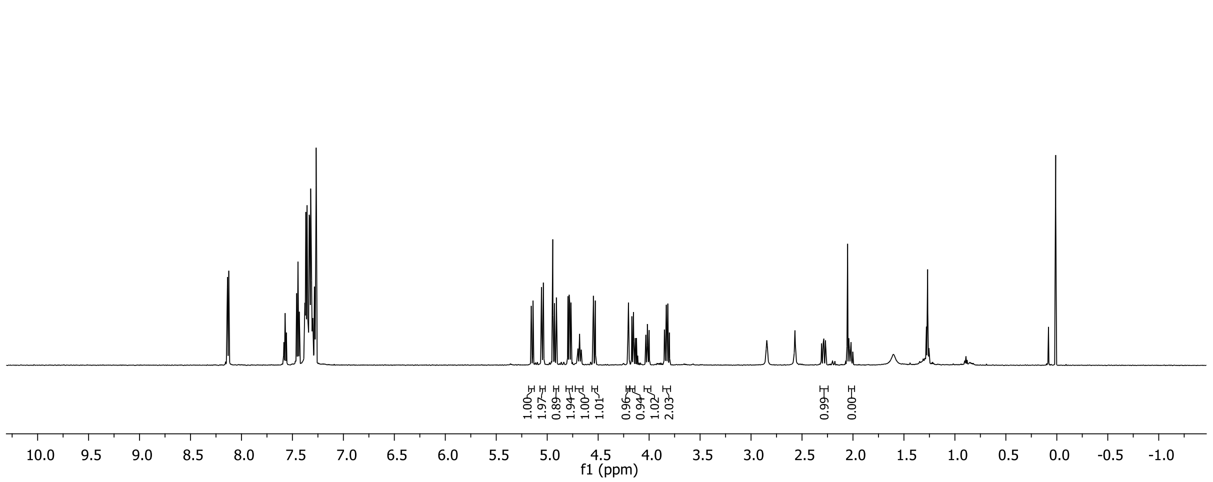

Compound **28**, ^{13}C NMR, 150 MHz, $CDCl_3$

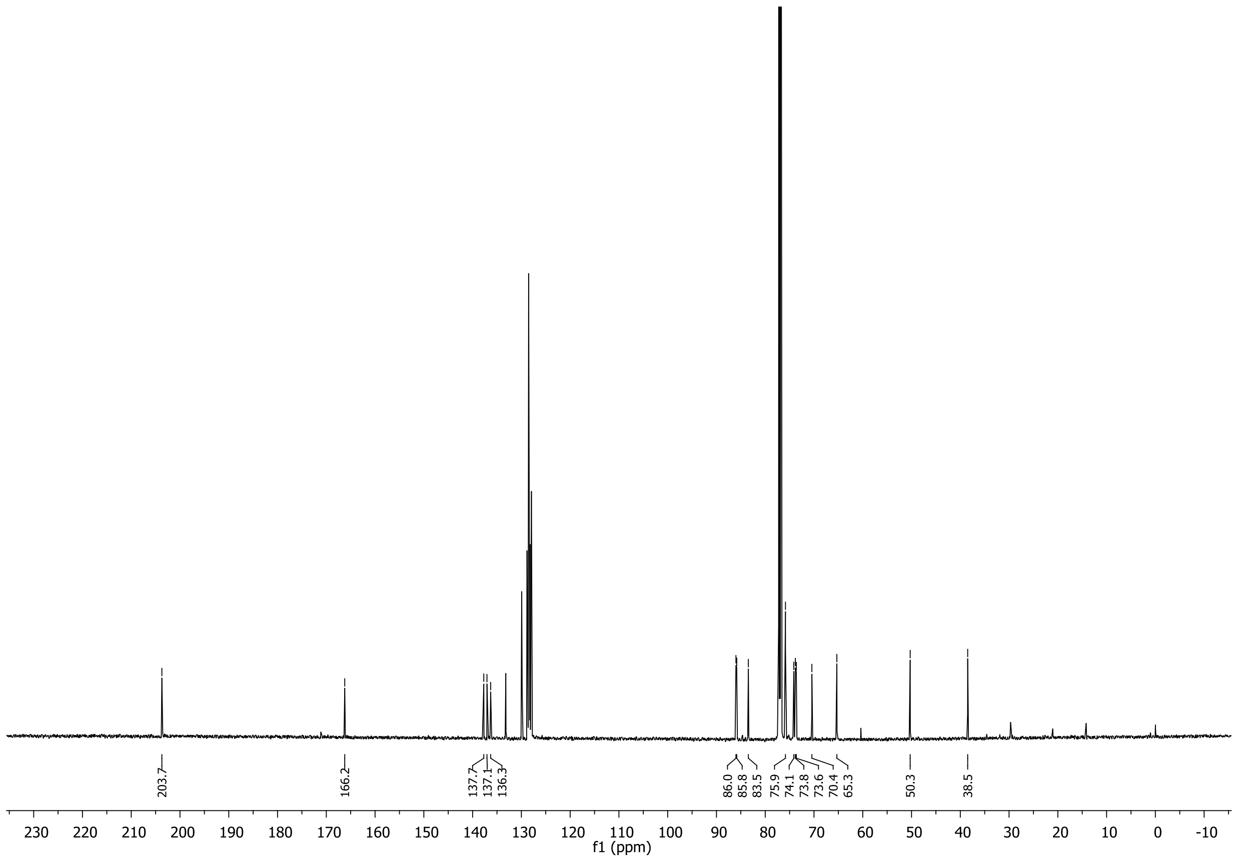

Compound **29**, ^{1}H NMR, 600 MHz, $CDCl_3$

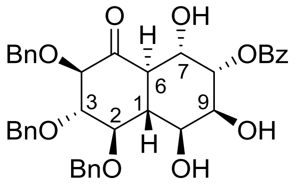

29

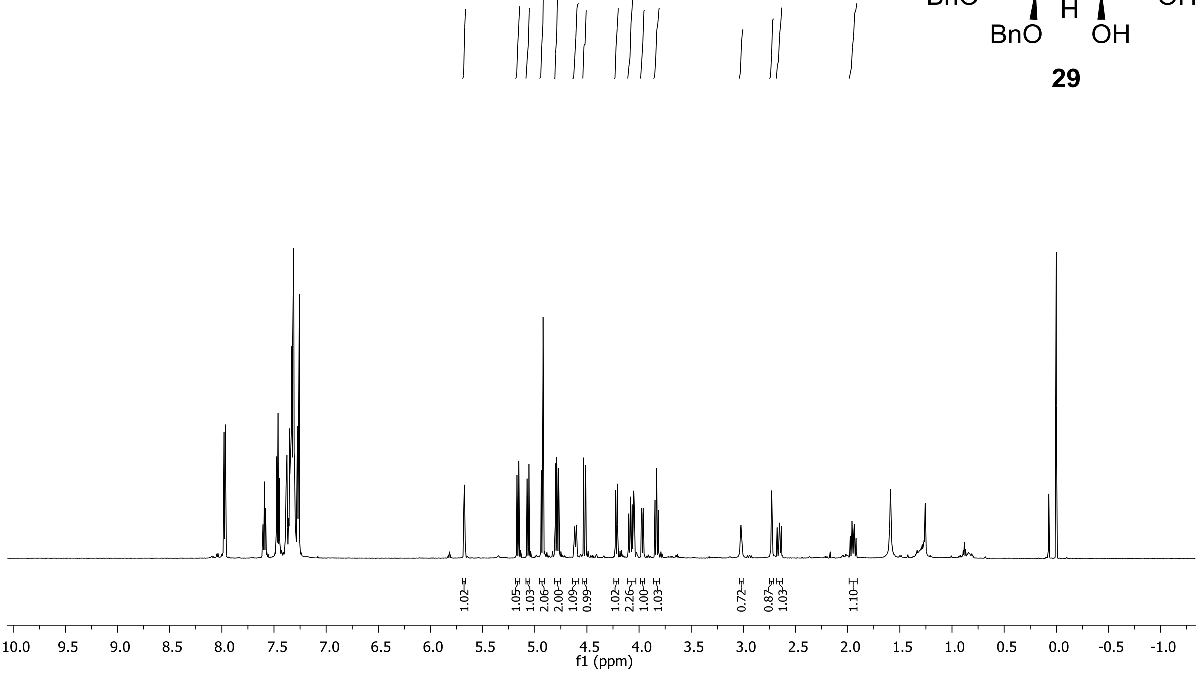

Compound **29**, ^{13}C NMR, 150 MHz, $CDCl_3$

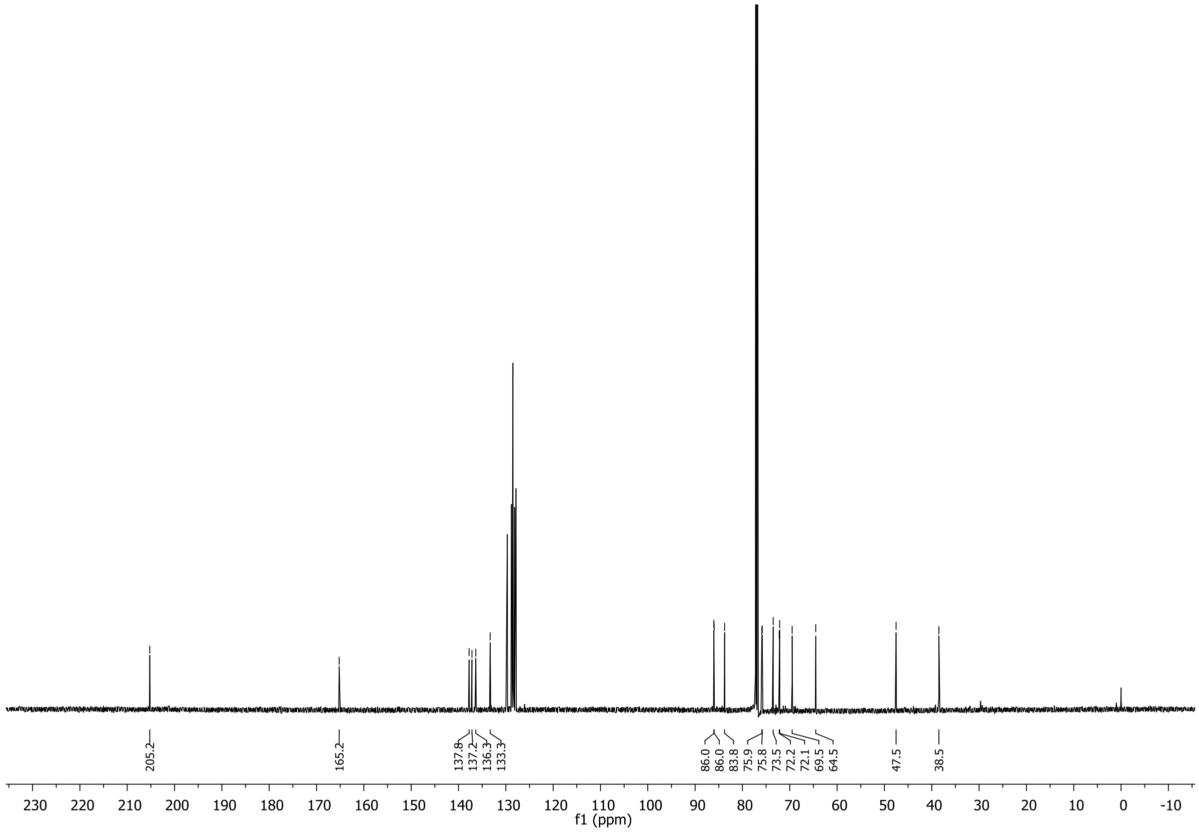

Compound **30**, ^{1}H NMR, 600 MHz, $CDCl_3$

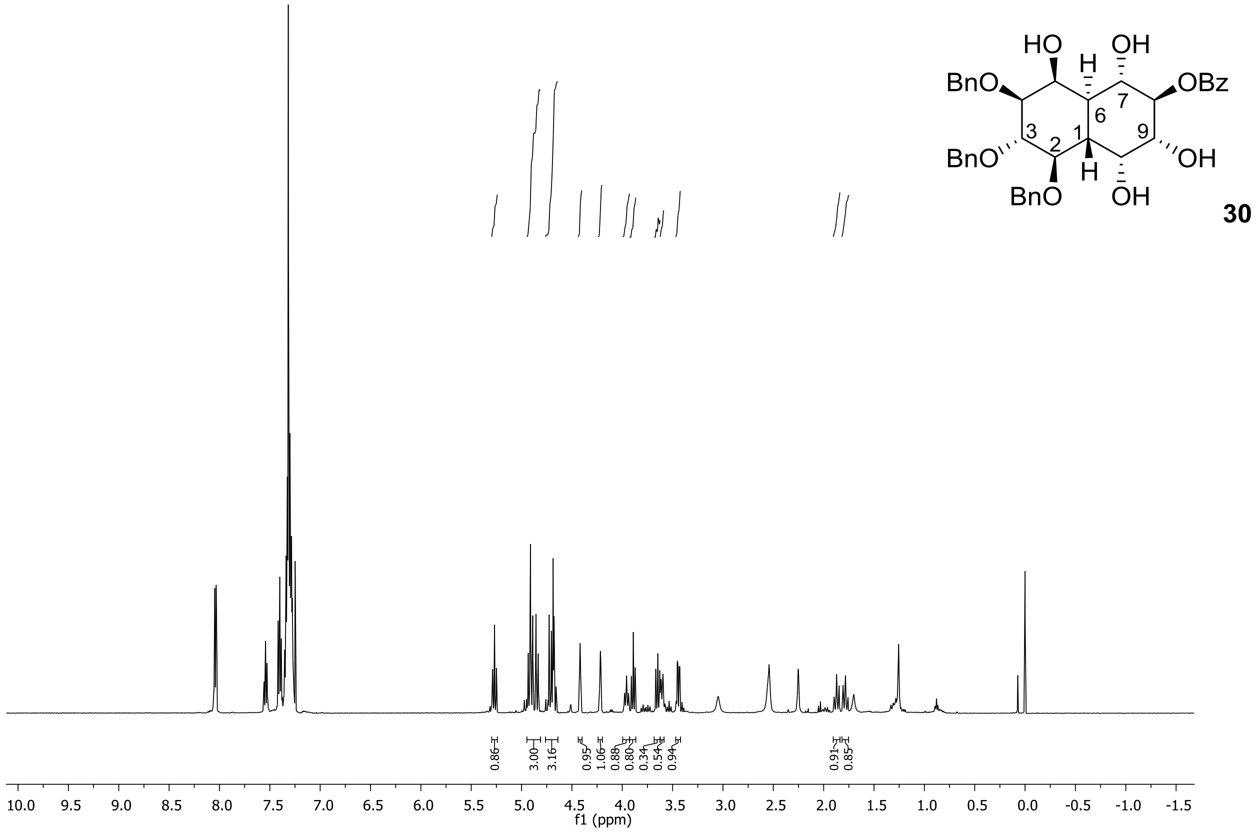

Compound **30**, ^{13}C NMR, 150 MHz, $CDCl_3$

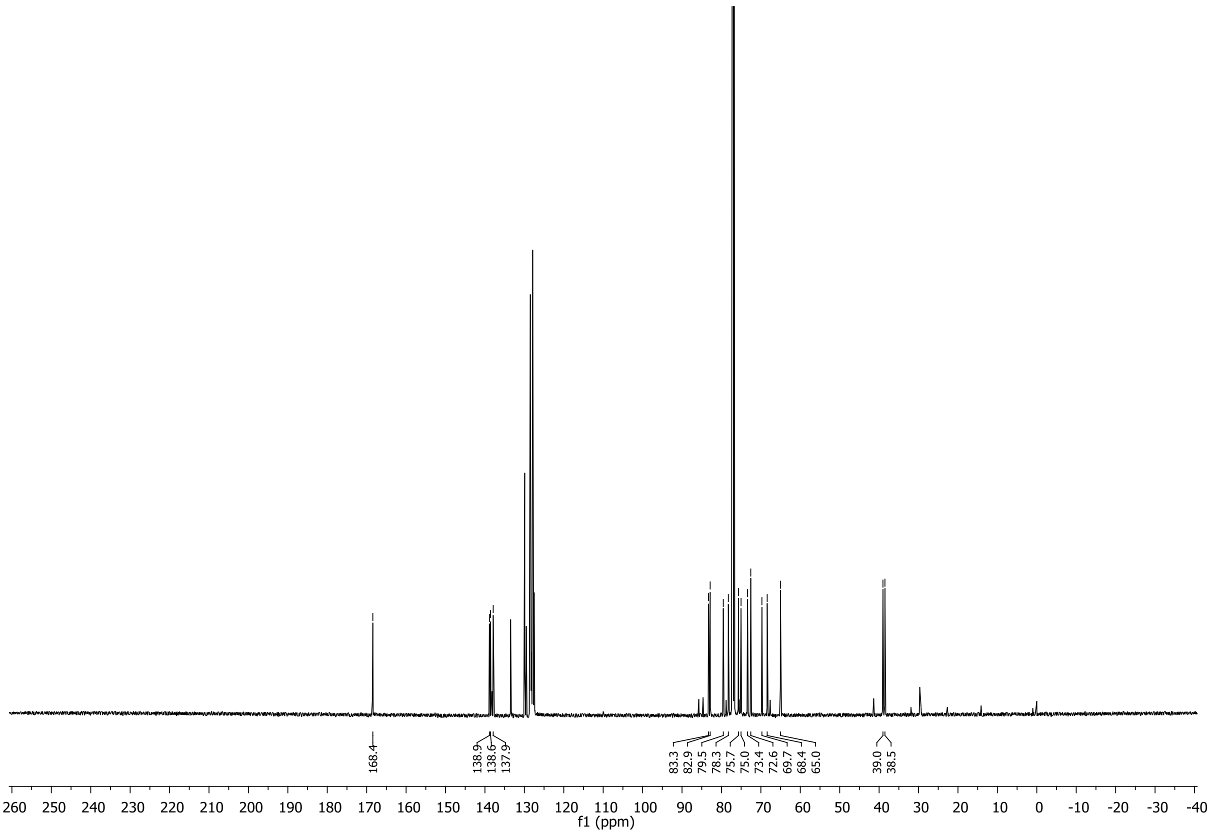

Compound **31**, ^{1}H NMR, 600 MHz, CDCl$_3$

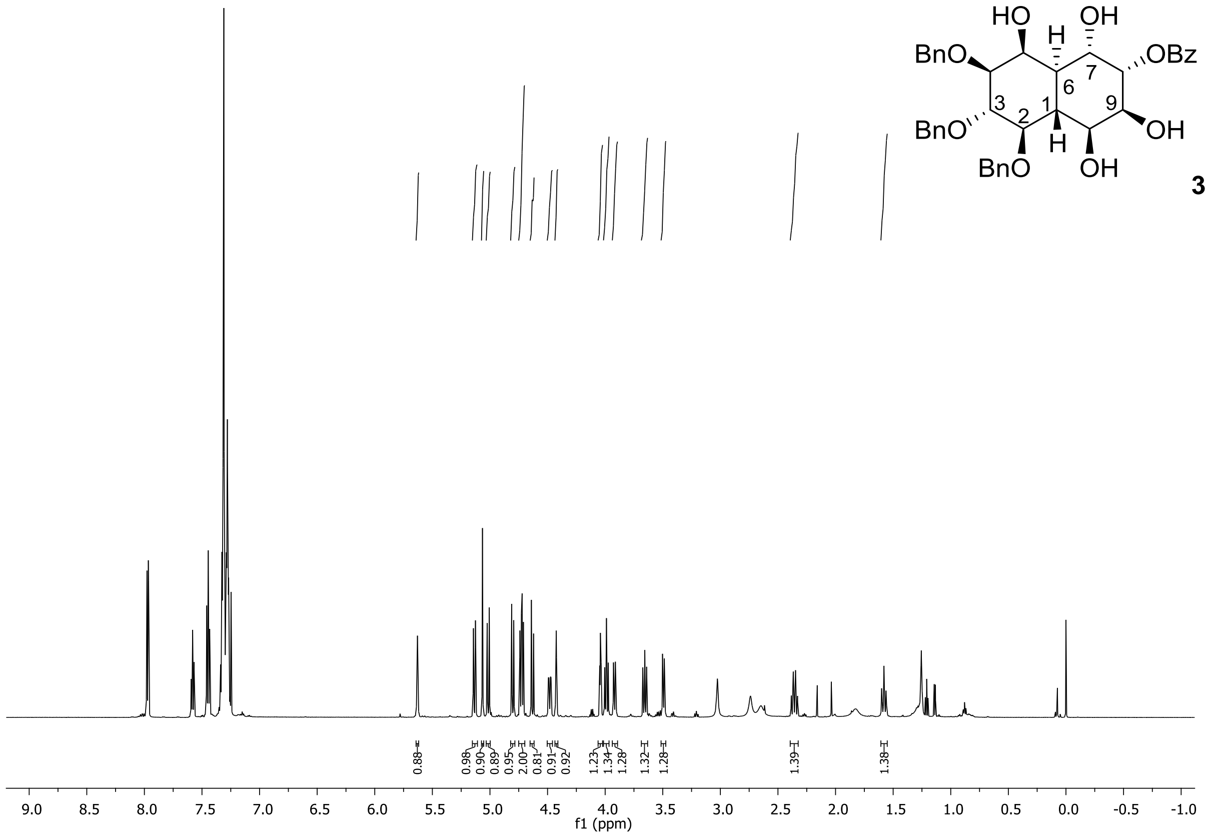

Compound **31**, ^{13}C NMR, 150 MHz, CDCl$_3$

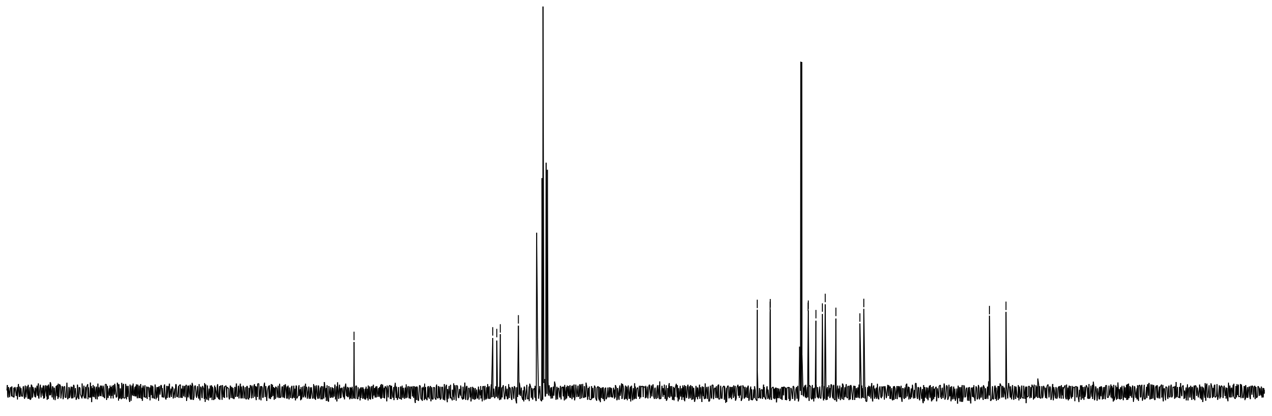

Compound **32**, ^{1}H NMR, 600 MHz, $CDCl_3$

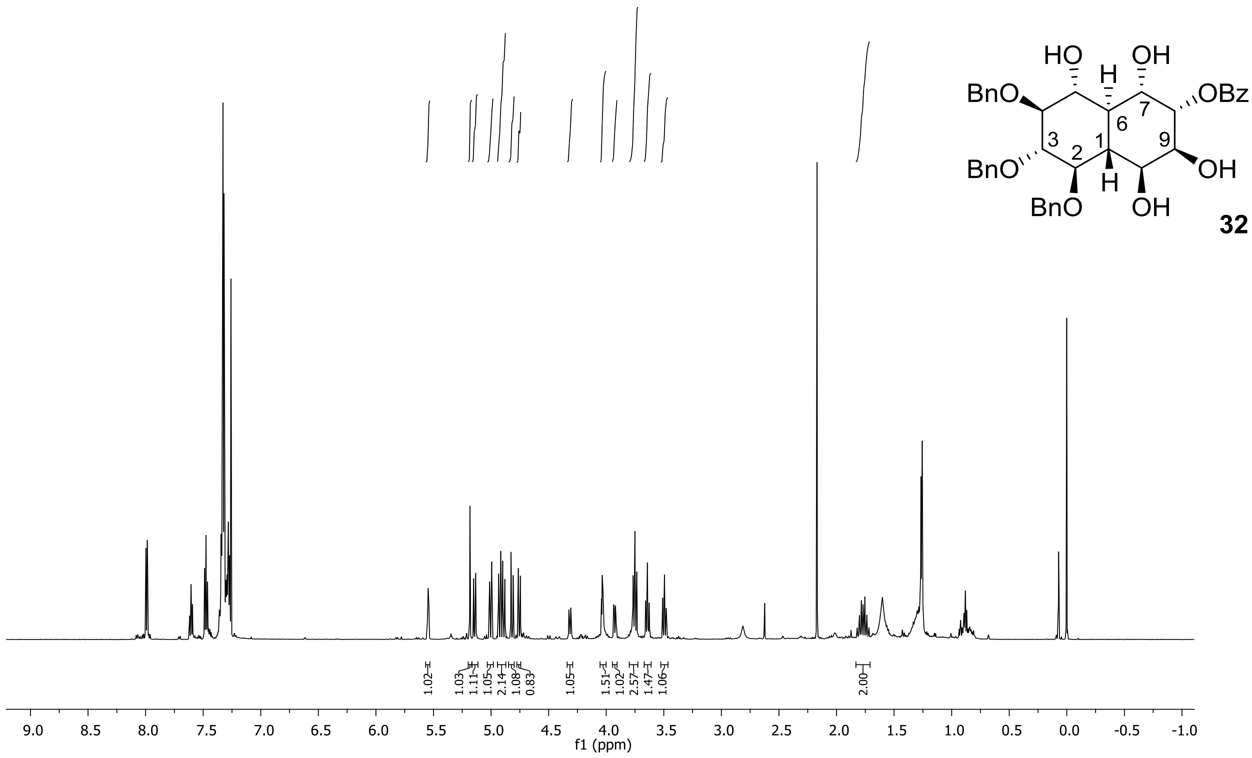

Compound **32**, ^{13}C NMR, 150 MHz, $CDCl_3$

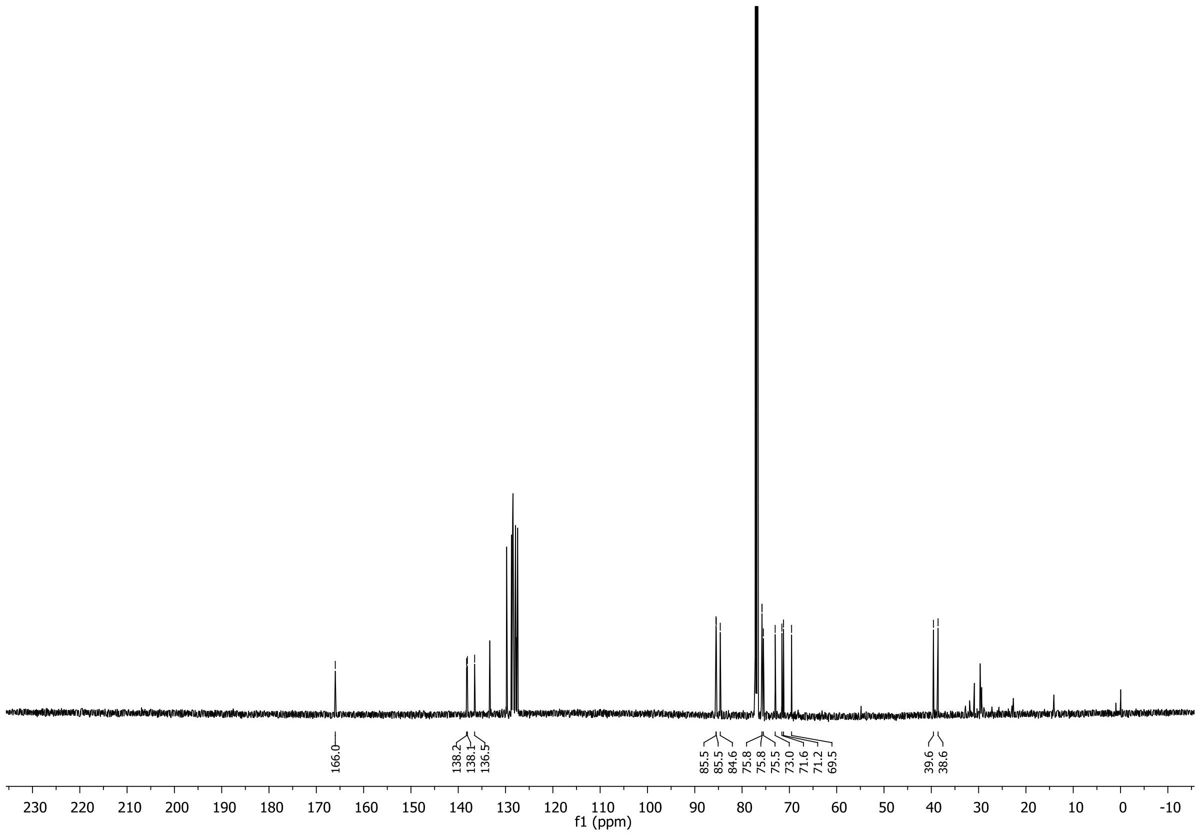

Supplement: File 1 — Experimental procedures, spectral data, and copies of the 1H and 13C spectra for all new compounds. [file Beilstein_J_Org_Chem-12-2602-s001.pdf]
